# Genetic and physiological mechanisms of grapevine resistance to *Xylella fastidiosa* infection: a progressive acquisition of knowledge

**DOI:** 10.3389/fpls.2026.1946516

**Published:** 2026-09-21

**Authors:** Serafina Serena Amoia, Pasquale Saldarelli

**Affiliations:** Consiglio Nazionale delle Ricerche, Istituto per la Protezione Sostenibile delle Piante (CNR-IPSP), Bari, Italy

**Keywords:** biotechnology, breeding, grapevine, MAS, Pierce’s disease, resistance, *Xylella fastidiosa* subsp. *fastidiosa*

## Abstract

Pierce’s disease (PD), caused by *Xylella fastidiosa* (*X. fastidiosa*), has challenged grape cultivation in the US since the late nineteenth century and has stimulated extensive exploration of grapevine genetic diversity for sources of resistance. Early observations made during the first outbreaks in California, when the disease was known as “Anaheim disease”, revealed marked variety-dependent differences in survival. Some varieties were proposed as potential top-grafts or rootstocks, reflecting the recognition of the heritable nature of resistance and its possible incorporation into newly developed varieties. Early resistant materials were primarily interspecific hybrids between *Vitis vinifera* and native southeastern American *Vitis* species, suggesting that the most effective resistance sources occurred outside the *V. vinifera* gene pool. Similar failures of grape cultivation in Florida were subsequently attributed to PD, and field trials confirmed the high susceptibility of European grape varieties, whereas several native grapes and muscadine species displayed intermediate tolerance or high resistance to the disease. From the 1950s onward, systematic screening and breeding programs identified multiple heritable resistance sources in native southeastern American grape species. Genetic studies indicated that resistance could depend on the inheritance of dominant alleles at multiple loci, explaining the low frequency of resistant progeny in some crosses. These findings prompted breeding programs aimed at introgressing resistance into elite bunch-grape backgrounds while retaining desirable fruit-quality traits. The identification of the bacterium “*Xylella fastidiosa*” as the causal agent of PD further facilitated screening and phenotyping through microscopy, serological and molecular assays, standardized inoculation protocols, and the combined evaluation of bacterial population size and symptom severity. A major advance was the identification of resistance in *V. arizonica*. Genetic mapping led to the discovery of the major resistance locus *PdR1* and associated molecular markers, enabling marker-assisted selection and accelerating the elimination of susceptible seedlings during breeding. More recently, transgenic approaches have complemented conventional breeding strategies. Overall, the available literature confirms the general susceptibility of *V. vinifera* to PD while highlighting the progressive enhancement of resistance through the exploitation of wild grape germplasm, conventional breeding, marker-assisted selection, and genetic engineering.

## 1 Taxonomic notes on the genus *Vitis* and on PD-associated *Xylella fastidiosa* strains

The genus “*Vitis*”, belonging to the family *Vitaceae* and formally established as a taxonomic genus, comprises approximately 90 species (in addition to numerous hybrids) mainly distributed across North America and East Asia (https://glossary.wein.plus/vine-systematics). Within this genus, two major evolutionary lineages have been recognized and are often considered subgenera: subgenus “*Vitis*” (frequently referred to as “*Euvitis*”), which includes the typical bunch grapes like *V. vinifera*, and subgenus “*Muscadinia*” Planch, which includes the muscadine grapes native to the southeastern USA. The two groups differ not only phylogenetically but also biologically, with *Vitis* species having 2*n* = 38 chromosomes, whereas *Muscadinia* species have 2*n* = 40 (Buck and Worthington, 2022). For this reason, more recently, phylogenomic studies suggested that *Muscadinia* may deserve recognition as a separate subgenus.

Although several *X. fastidiosa* subspecies have been described worldwide, Pierce’s disease (PD) of grapevine is primarily caused by *X. fastidiosa* subsp. *fastidiosa*, particularly sequence types (ST)1 and ST2. These strains have been responsible for most historical and contemporary PD outbreaks in viticultural systems and represent the main pathosystem investigated in studies of grapevine resistance.

## 2 First evidence of resistant grape species

Evidence of different varietal responses to PD emerged from its first description as “Anaheim Disease”, named after the Californian town where the disease was first observed (Gardner and Hewitt, 1974). Early field observations carried out by Newton B. Pierce in the Sacramento Valley identified interspecific hybrids such as Lenoir (*V. aestivalis* x *V. vinifera*) and Isabella (*V. labrusca* × *V. vinifera* “Petit Meslier”) as more resistant than *V. vinifera* varieties, which were generally highly susceptible and frequently collapsed within a few years after planting. The new planting experiences of *V. vinifera* varieties in the southeastern USA, particularly in Florida, failed completely, thus prompting the Federal Government to carry out field tests and laboratory investigations, which led to the conclusion that the grape degeneration observed in Florida was the same as that reported in California (Stoner et al., 1951; Stoner, 1953; Stover, 1960). A similar decline was reported in Costa Rica, where some *V. vinifera* and *V. labrusca* varieties did not survive the disease, while *V. indica* (synonym *V. caribaea*) and other Florida-native species were able to survive (Goheen et al., 1979). From these first experiences, it was observed that *V. vinifera* varieties collapsed within two years of planting, while *V. simpsonii*, *V. labrusca*, and *V. lincecumii* and their interspecific crosses showed different levels of resistance, highlighting the remarkable resilience of native southeastern US species, where PD was widespread. Field trials also demonstrated that muscadine grapes (*V. rotundifolia* Michx, also called *Muscadinia rotundifolia*) were more tolerant than *V. vinifera*, which remained highly susceptible (Loomis, 1958, 1968; Nesbitt et al., 1976; Overcash et al., 1982). Some years later, Mortensen and collegues observed resistance in three *Vitis* species (*V. aestivalis* ssp. *smalliana* Bailey, *V. simpsonii* Munson, and *V. shuttleworthii* House) and their controlled crossings under natural disease pressure. They posited that the resistance was due to the complementary action of three dominant genes: the offspring must inherit a dominant allele at every locus to be resistant, but when the loci assort independently, only a small number of resistant progenies are obtained from these crosses (Mortensen, 1968, 1971). Mortensen and Stover (1990) identified six *V. aestivalis* and *V. shuttleworthii* clones potentially useful for producing new PD-resistant varieties. They also indicated a set of susceptible *V. vinifera* varieties for use in crossing, based on their preeminent features of seedlessness, early ripening, fruit size, and high quality. Consolidated observations (Hopkins et al., 1974b; Mortensen et al., 1977; Roger and Mortensen, 1979) of resistance in the subgenera *Euvitis* and *Muscadinia* promoted the initiation of a breeding program to introgress resistance traits into *V. vinifera*, which led to the release of the first PD-resistant variety named Orlando Seedless, a *Vitis* interspecific cross with *V. smalliana* parentage (Mortensen and Gray, 1986). The severe impact of PD also in the Tropics pushed local researchers to search for and breed for resistance (Jimenez and Ingalls, 1990). *V. caribaea* plants growing in the forests, near declining vineyards, were found to be unaffected by the disease, as were their F1 hybrids (*V. caribaea* × *V. vinifera*), likely confirming Mortensen’s theory that resistance is controlled by dominant genes.

Mortensen and Stover (1990) summarized the first 40 years of breeding research by identifying a set of “…useful varieties that can be grown productively in areas formerly considered unsuitable for grape production”. A detailed history of the breeding efforts conducted in the southeastern USA, with a focus on tolerance of muscadine grape, was reported in Stafne et al. (2015). After describing the characteristics of native southeastern species, the authors compiled an extensive list of past and future bunch grape and muscadine breeding programs in that area, pointing out the main challenges and limitations.

## 3 *Muscadinia rotundifolia* as a source of PD tolerance

The discovery of the PD-associated “rickettsia-like organism” in the xylem, as a proxy for PD infection (Goheen et al., 1973; Hopkins and Mollenhauer, 1973; Auger et al., 1974), fueled studies aimed at characterizing the ultrastructural alterations of infected grapevines (Mollenhauer and Hopkins, 1974). The observed aggregates were found to hamper the flow of water and mineral nutrients in the xylem vessels of infected grapevines (Mollenhauer and Hopkins, 1976), a phenomenon also characterized by the formation of tyloses and gums, a plant reaction already described in response to the presence of a vascular pathogen (Hopkins and Mollenhauer, 1975).

Among the grape species investigated, muscadine grapes emerged as one of the most valuable sources of PD tolerance. Microscope observations revealed that muscadines were not immune to this “organism” and harbored few cells *per* infected xylem vessel. However, the extent of plant response to counteract the infection appeared to be dependent on their different genetic backgrounds (levels of inbreeding) (Hopkins et al., 1974a) and was found to be particularly pronounced in the tolerant muscadines Pride and Scuppernong as well as in the highly tolerant *V. munsoniana* Simpson. Moreover, muscadine accessions originating from Georgia showed a lower PD incidence than those developed in North Carolina or Mississippi, suggesting the existence of substantial intraspecific variation in PD tolerance (Hopkins et al., 1974b). Additional evidence was provided by field and greenhouse studies. Hopkins and Thompson (1984), during a survey carried out in Florida, reported that vascular occlusions and leaf marginal necrosis appeared two months earlier in susceptible *V. labrusca* Schuyler than in tolerant *V. rotundifolia* varieties Carlos and Welder. Some years later, an artificial inoculation carried out by Hopkins (1989) confirmed the tolerance of Carlos, showing that the upward migration of bacterial cells along the stem was rapid during the first 15 days after inoculation, but became markedly slower in the following weeks of infection, resulting in the development of mild foliar symptoms. Therefore, he posited that the lower rate of multiplication and the reduced colonization of vessels could be associated with the presence of physical barriers (i.e., obstruction of the vessel) and the antimicrobial activity of gums and tyloses produced by the host plant. Further insights were obtained through electron microscopy. Comparing artificially inoculated susceptible bunch grapes and tolerant muscadines, Pi-Yu Huang and Milholland (1986) observed differences in the morphology of bacterial cells in the xylem vessels of the two species, thus proposing a possible relationship between the plant resistance and the phenotype of the bacterial cell wall. In muscadine grapes, cells appeared smooth-walled and encapsulated in an electron-dense, pectin-based matrix derived from the plant, which could help the grapes defend against the pathogen. By contrast, in susceptible grapes, bacterial cells had rippled cell walls.

Additional evidence of the variation in PD tolerance among muscadine accessions was described by Fry and Milholland (1990a). They found that symptom expression was more severe in artificially inoculated French Colombard than in Carlos and Noble, the latter two of which were classified by the authors as resistant and tolerant. Indeed, French Colombard hosted, respectively, 10- and 100- to 1,000-fold greater numbers of bacterial cells in petioles and stems, respectively. Moreover, in this variety, a lower proportion of aggregates that clog the vessels, favoring further movement of bacterial cells, was also observed by Lane (1997). Parallel microscope observations demonstrated that the percentage of leaf vein vessels occluded by bacterial cells and pectin was higher in French Colombard than in the two muscadine varieties (Fry and Milholland, 1990b), while the production of tyloses was similar in all the varieties considered.

## 4 Early approaches for assessing bacterial population and host colonization

Early investigations of *X. fastidiosa* colonization of vessels relied primarily on microscopy and serological assays, which provided the first evidence of genotype-dependent differences in bacterial multiplication and systemic movement. Since the 1980s, the production of an antiserum specific to *X. fastidiosa* and the development of serological assays have allowed for a massive campaign of artificial inoculations to screen as much grape germplasm as possible. Raju and Goheen (1981) vacuum-infiltrated 25 varieties largely cultivated in Napa Valley, California, and after a phenotypic screening, they ranked plants according to the concentration of bacteria in the leaf petioles estimated by enzyme-linked immunosorbent assay (ELISA). The varieties Sylvaner and Chenin Blanc were the least affected, while French Colombard and Sauvignon Blanc had the highest bacterial population levels.

Rootstock varieties also differentially affect the severity of symptoms of grafted scions, an effect already observed by Pierce in his first description of “Californian vine disease” and experienced in the southeastern US area, where hot and humid conditions favor the prevalence of PD (Mortensen et al., 1994). Fishleder and Walker (1999) extended the screening to 18 rootstocks, five interspecific hybrids, and more than 100 accessions belonging to 24 grape species originating from the southern USA and Mexico, confirming the absence of PD symptoms within the *Muscadinia* subgenus and in *V. rufotomentosa* plants (genetically associated with *V. aestivalis*) and in the rootstock St George. Lu et al. (2008) evaluated the performance of 10 major grape rootstocks in Florida in a long-term field trial, observing that Ramsey, St George, 5C, and 110R showed fewer PD symptoms than Freedom, 3309C, and O39-16, the latter three of which had 50% of plants die in the third growing season. Ramsey was the only rootstock variety whose plants survived up to the sixth growing season. Therefore, the authors recommended St George, Stover, Ramsey, 110R, and 5C as suitable grape rootstocks in the Florida climate.

Greenhouse trials allowed for assessing the greater susceptibility of the *V. vinifera* variety Red Globe than that of Thompson Seedless, with only 2%–7% of Red Globe plants surviving the infection, compared with 51% of Thompson Seedless at 30 weeks postinoculation (Krell et al., 2008). A survey carried out on more than 60 varieties conserved at the National Clonal Germplasm Repository (NCGR) in Davis, California, further confirmed that the majority of accessions collected from the southeastern USA are effectively resistant to PD, although some of them develop a systemic *X. fastidiosa* infection (Stover et al., 2008). A final assessment of field studies, based on cumulative yield per plant from 2011 to 2017, found that varieties Favorite and Villard Blanc gave the highest yield, while Conquistador gave the lowest (Coneva et al., 2019). Based on the results obtained, considering their tolerance to PD and the combination of fruit yield, cluster number, and berry size, Villard Blanc, Black Spanish, and Favorite were considered the best sustainable choices for commercial grape production in Alabama and the southeastern USA.

## 5 Grapevine xylem anatomy and pit membrane composition shape host resistance to PD

The effects of *X. fastidiosa* infection on the colonization and occlusion of petioles and stem vessels were evaluated in four grape genotypes supporting different extents of bacterial growth (Fritschi et al., 2008). Using scanning electron microscopy (SEM), the greatest number of occluded vessels was observed in Chardonnay, while the fewest were found in *Vitis arizonica/candicans* b43-17, which did not significantly differ from the other two species, *V. aestivalis* var. *smalliana* and var. *rufotomentosa*. The frequency of occluded vessels was directly correlated with bacterial loads assessed by ELISA. Conversely, tylosis formation was strongly induced by the infection in Chardonnay and *V. aestivalis* var. *smalliana* but not in *V. arizonica/candicans* b43-17, supporting the idea that resistance mechanisms based on xylem vessel occlusion in the resistant genotype were not involved and indicating that hyper-tylosis induction in infected Chardonnay may be detrimental to symptom expression. The xylem anatomy of varieties with different degrees of susceptibility was inspected by introducing air and paint into the vessels. The tolerant variety Sylvaner presented smaller stem vessel diameters (between 85 and 100 µm) and 20% more parenchyma rays than the susceptible Chardonnay (150 and 300 µm, respectively), possibly restricting bacterial movement and emboli formation (Chatelet et al., 2011). The extent of vessel colonization in susceptible and resistant grapes was tracked using a green fluorescent protein (GFP)-tagged *X. fastidiosa*: the bacterium colonized fewer vessels and reached lower population sizes in the stems of the resistant varieties Roucaneuf and Tampa (both *Vitis* interspecific hybrids) than in the susceptible varieties Cabernet Sauvignon and Chenin Blanc (Baccari and Lindow, 2011). On the contrary, comparable bacterial populations and similar proportions of colonized vessels were observed in the petioles of all varieties. Therefore, based on these results, the authors concluded that the resistance is unlikely to be associated with the presence of antimicrobial compounds in the xylem sap since these compounds should be equally distributed along the vascular system. A more convincing hypothesis involves a resistance mechanism capable of counteracting the activity of the cell wall-degrading enzymes (CWDEs) produced by the bacterium. Immunohistochemical analysis revealed that intervessel pit membranes (PMs) of PD-resistant hybrids *V. arizonica* × *rupestris* (89-0908) and *V. vinifera* × *arizonica* (U0505-01) lacked fucosylated xyloglucans (XyGs) and weakly methyl-esterified homogalacturonans (HGs), which were highly present in PMs of PD-susceptible Chardonnay and Riesling (Sun et al., 2011). Moreover, occlusions caused by tylosis formation blocked > 60% of vessels across internodes in the two susceptible varieties, resulting in a *ca*. 92% loss of specific hydraulic conductivity upon infection; by contrast, only *ca*. 20% of vessels of the PD-resistant accessions appeared occluded, mainly localized near the inoculation site and affecting one-third of the hydraulic conductivity. The extensive blockage observed in susceptible plants was not sufficient to stop the systemic spread but instead severely reduced the water flow, thereby exacerbating the development of symptoms and contributing to vine decline (Sun et al., 2013).

Differences in the radial connectivity of xylem vessels of susceptible and resistant grapevines were observed by Brodersen et al. (2013), who focused on the so-called vessel relays, an anatomically distinct class of narrow-diameter vessel elements that are radially oriented and connected to the large xylem vessels. A greater proportion of vessel relays (*ca.* 26%) was found in Chardonnay than in the resistant *V. arizonica*. Such anatomical differences may cause a higher propensity of susceptible varieties to embolism-triggered water stress and the spread of xylem-dwelling pathogens, which, coupled with chemical differences in the composition of PMs between these two grape species, may contribute to the establishment of PD infection. Indeed, Chardonnay proved to be significantly more susceptible than Cabernet Sauvignon, likely due to differences in the vulnerability of PMs to CWDE, associated with differences in the amounts of fucosylated XyGs (Ingel et al., 2019). These polysaccharides, which constitute the PMs’ surface, are targeted by the *X. fastidiosa* β-1,4-endoglucanases, whose loss by mutation reduces bacterial virulence, population size, and the progression of symptoms.

The hypothesis that anatomical and chemical differences contribute to the susceptibility was also explored by Deyett et al. (2019) in different *V. vinifera* varieties. Thompson Seedless was the most susceptible, having both the highest pathogen population and the most severe symptoms at 12 weeks postinoculation. The concomitant loss of *ca.* 90% of stem hydraulic conductivity and significant vessel occlusions demonstrated that anatomical features shape the plant response to the bacterium. In this ranking, the wine grapes Cabernet Sauvignon and Merlot and the table grape Scarlet Royal were less susceptible than Thompson Seedless.

To provide new insight into the influence of CWDE on the xylem conduits and investigate how the enzymatic degradation of PMs affects the hydraulic conductivity, Fanton and Brodersen (2021) perfused the stems of six varieties, including the resistant genotypes *V. arizonica/candicans* b43-17, Hybrid 502-20 (an 88% *V. vinifera* cv Chardonnay), Lenoir, Blanc du Bois, and the susceptible Syrah and Chardonnay, with a polygalacturonase (PG) solution, thus simulating PM degradation in the absence of the bacterium. PMs were less degraded by PG enzymes in b43-17and Hybrid 502-20 than in the other genotypes tested. Rather than increasing water transport, the enzymatic treatment generally decreased the hydraulic conductivity, with the strongest drops observed in Blanc du Bois (*ca*. 50%) and Syrah (*ca*. 40%) and the smallest in Lenoir (*ca*. 6%). At the same time, the treated stems became more vulnerable to embolism, since the median air-seeding pressure shifted toward less negative values in all genotypes, indicating weaker PMs. Microscope observations showed that the damaged PMs had localized pore enlargement or partial membrane degradation, and although the damaged area was small (*ca*. 2%–9% of the whole pit-aperture area), it was sufficient to compromise the entire hydraulic system. Importantly, these effects occurred in the absence of tyloses or gel formation, suggesting that PM breakdown caused by PGs alone can impair hydraulic conductivity and increase the likelihood of air entry between vessels. Moreover, in PD‐susceptible genotypes, the reduction in both hydraulic conductivity and stomatal conductance, in turn, led to a decline in photosynthetic efficiency and, as a consequence, to a depletion of starch content in the vessels (Fanton et al., 2022). The same varieties were also subjected to high-resolution X-ray microcomputed tomography to test the hypothesis that the anatomical traits facilitating *X. fastidiosa* spread could explain the different degrees of susceptibility among the different varieties. It was found that radial (i.e., intra-ray sectors) or tangential (i.e., across ray sectors) vessel connectivity, measured by intervessel pit area (Ai) or intervessel contact fraction (Cp), did not explain differences in PD resistance among the genotypes, as both traits are highly conserved in the tested resistant and susceptible genotypes. The results obtained refuted the mechanism of compartmentalization of the pathogen based on vessel diameters, in favor of an active host biochemical response to the bacterium (Fanton et al., 2024).

## 6 From resistance discovery to genetic mapping

### 6.1 First breeding efforts to introgress PD resistance

The consolidated evidence that muscadines are tolerant to PD and to the main grape diseases, such as downy mildew and anthracnose, has promoted extensive research aimed at introducing these resistance traits into *V. vinifera* by interspecific crosses while preserving the superior quality of bunch grapes. However, beyond the need for several generations of backcrossing, the major obstacle to obtaining such hybrids is the difference in chromosome number between the parental species (*V. vinifera* = 38 and *V. rotundifolia* = 40), coupled with the existence of morphological and cytological features that hinder flower fertilization and the survival of the hybrids. Some attempts to cross the two species using *V. vinifera* as the female parent led to the formation of hybrids that were later shown to result from contaminated pollination involving *V. vinifera* × *V. vinifera* rather than the intended *V. vinifera* × *V. rotundifolia* crosses (Lu, 2000; Lu et al., 2000). The opposite crossings, using *V. rotundifolia* as the female parent, generated only two hybrids (out of 20,000 flowers pollinated) with intermediate characteristics, including PD resistance, between the two subgenera, thus confirming Mortensen’s hypothesis that resistance genes are dominant. To overcome the obstacle of interspecific crossing, Krivanek and Walker (2000) produced fertile hybrids of *V. rupestris* × *V. rotundifolia*, which were subsequently crossed with the Chardonnay plants. This sort of “genetic bridge” would overcome species incompatibility, thus allowing the production of large seedling populations with *V. vinifera* parentage that could be screened to identify DNA markers linked to the resistance.

We need to look back to 1961, when Harold P. Olmo, a grape breeder and professor at the University of California, Davis, collected 85 genotypes during a trip to Mexico, where PD is also endemic, including a few clones of *V. arizonica* (Vasquez, 2006). Following the experiences of colleagues in the southeastern USA, Olmo crossed female *V. rupestris* with male *V. rotundifolia* individuals, obtaining an F1 population, named “89”, that showed strong resistance to both the dagger nematode *Xiphinema index* (the vector of Grapevine fanleaf virus) and PD, with both traits segregating as single loci. To genetically characterize the locus carrying the resistance trait, Walker, who inherited the “89” group, crossed two siblings, D8909–15 and F8909-17, to generate an F2 population later named “9621” (Doucleff et al., 2004). Successive studies using single sequence repeats (SSRs) markers and morphological features (i.e., neither *V. rupestris* nor *V. rotundifolia* has hairs on their stems or leaves, and their progeny should be sterile, which was not the case for the 89 and 9621 populations) revealed that: (1) D8909–15 and F8909–17 were not siblings; (2) most of the resistant “89” seedlings originated from *V. rupestris* females unintentionally pollinated by *V. arizonica* and its hybrids with *V. candicans*; and (3) D8909–15 and F8909–17 were, respectively, interspecific hybrids of *V. rupestris* “A. de Serres” × b42-26 (a *V. arizonica* selection from Baja California, Mexico) and *V. rupestris* “A. de Serres” × b43-17 (a *V. arizonica* from Monterrey, Mexico) (Riaz et al., 2007). Therefore, only very few seedlings had the *V. rupestris* or *V. rotundifolia* parentage that Olmo intended to obtain. Nevertheless, the unexpected discovery of the PD resistance in *V. arizonica* prompted Walker and collaborators to direct future investigations toward this grape species.

### 6.2 Development of the first genetic map for PD resistance

A genetic linkage map of the “9621” population, accounting for 475 markers, distributed by amplified fragment length polymorphism (AFLP), SSR, and random amplified length polymorphism (RFLP), was compiled. Markers were ordered in 17 and 19 linkage groups, respectively, of the maternal D8909–15 and paternal F8909-17 (Doucleff et al., 2004). Although only moderately saturated, this map represents the first genetic tool to identify the PD resistance genes. To have a better chance to discover markers for marker-assisted selection (MAS) for expediting the breeding process and the screening of grape germplasm, Reis et al. (2002) tested 120 seedlings, derived from crosses between the resistant 8909-08 (*V. rupestris* x *M. rotundifolia*) and four susceptible USDA table grape selections. To go in depth into the genetic of *V. arizonica* resistance, Krivanek et al. (2005a) established a factorial mating design using eight genotypes of the “8909” population, consisting of interspecific hybrids of susceptible *V. rupestris* “A. de Serres” as female parent and b42-26 (*V. arizonica*, genotype D-8909-15, resistant), Y14-122 (*V. arizonica* × *V. vulpina*, genotype J8909-02, resistant), b43-17 (*V. arizonica/candicans*, genotypes F8909-1, F8909-8, F8909-16, and F8909-26, resistant), and variety Pillans (*V. rupestris*, genotypes C8909–07 and C8909-19, susceptible) as male parents. Disease progression of the obtained 16 full-sib populations artificially inoculated with *X. fastidiosa* suspension was scored using three parameters: leaf scorch (LS), a cane maturation index (CMI), and an index combining CMI with shoot stunting (CMSSI). Furthermore, quantitative assessments of the *X. fastidiosa* populations in stem tissues were obtained by ELISA.

### 6.3 Large-scale germplasm screening of grapevine germplasm for PD resistance

The need to evaluate several progeny plants persuaded Walker and coworkers (Krivanek et al., 2005b) to fine-tune a 5- to 7-month-long protocol of artificial inoculation and scoring for PD resistance in greenhouse conditions. The previously reported CMI demonstrated a high correlation (*R*^2^ = 0.91) with the performance of a set of field-characterized susceptible and resistant genotypes, while microscope observations of tylosis occlusions in the stems did not properly correlate. Conversely, the LS index showed a moderate correlation with field performance (*R*^2^ = 0.51), owing to the greater sensitivity of some genotypes to environmental stresses and the consequential low adaptation to greenhouse conditions. The authors recommended that the greenhouse trials be carefully conducted by inoculating one month before cane maturation and under a proper water regime. As confirmation of the need to conduct such trials carefully, Baumgartel and Walker (2007) observed that changing the size of the pots and reducing the irrigation volume could affect the response of resistant plants. However, besides the phenotype parameters (CMI and LS), ELISA values in greenhouse conditions proved to be highly correlated (*R*^2^ = 0.97) with the corresponding field performance of the tested genotypes. Significant differences in bacterial population levels between stem and leaf tissues were found in resistant genotypes, while the same was not true for susceptible ones (Buzkan et al., 2003; Krivanek and Walker, 2005). *Vitis* genotypes used in these studies to validate the protocol were the susceptible Chenin Blanc and Chardonnay and the resistant Blanc du Bois (background genotype *V. aestivalis* subsp. *smalliana* and *V. simpsonii*), Roucanef (background genotype *V. cinerea* and *V. berlandieri*), Tampa (background genotype *V. aestivalis* subsp. *smalliana*), Zehnder 71-50-1 (background genotype *V. aestivalis* subsp. *smalliana* and *V. simpsonii*), D8909-15 (background genotype *V. arizonica*), and D’Arpa (background genotype *V. shuttleworthii*). The major conclusion of this impressive research was that resistant genotypes host smaller *X. fastidiosa* populations and were able to limit bacterial systemic spread. In parallel, the resistance of native grapevine species and their hybrids continued to be assessed in the southeastern USA. Comparing ELISA data and crop yield over a 10- to 15-year period, Harkness and Morano (2004) reported the performance of hybrids in the Gulf Coast region of Texas. The strict link between bacterial population size and disease severity was confirmed, as Cynthiana (*V. aestivalis* × *V. vinifera*) hosted higher *X. fastidiosa* levels with a consequent lower yield than the tolerant Blanc du Bois and Black Spanish (*V. aestivalis* × *V. vinifera*, synonym Lenoir). To incorporate a broad range of PD-resistance sources into high-quality *V. vinifera* backgrounds, Walker and Tenscher (2005) tested in open field ca. 2,000 seedlings each year, evaluating their high-quality fruit juice and PD resistance. A group of promising highly resistant selections (0028-35, 0078-02, 0058-23, DC1-39, and 0028-44) was identified.

Since field trials and artificial inoculations require a long time, considerable space, and effort, Park et al. (2017) tested cell-free culture filtrates of *X. fastidiosa* as a rapid proxy assay for screening grape varieties. The authors applied the sterile filtrates of bacterial suspension to wounded leaves and measured the size of the necrotic halo caused by the toxic secondary metabolites produced by the bacterium. The test was largely consistent with resistance/susceptibility to PD previously observed in the field or in the greenhouse. Some discrepancies were attributed to different *X. fastidiosa* strains used in past experiments. Internal variability among the 51 tested muscadines was observed: the variety Doreen appeared to be the most susceptible, followed by African Queen, Higgins, and PAM, which showed intermediate susceptibility, while the other 18, including Pride and Noble, were found to be fully resistant. Among bunch grapes, Barbera, Blanc du Bois, and Cynthiana exhibited the largest necrotic halos; the other 20 tested varieties were moderately susceptible, and the remaining eight did not react to the filtrate. Moreover, Baumgartel and Walker (2007) further refined the greenhouse protocol by testing different growing conditions and inoculum concentrations, concluding that an inoculum of 10^7^ CFU/ml resulted in the highest infection success, while cutting back vines to two buds before inoculation reduced the bacterial population in the stem. The protocol was further optimized using different genotypes, plant tissues, and inoculation-site position by Fritschi et al. (2007a). Stem and leaf tissues of 18 *Vitis* genotypes were tested by ELISA at 34, 77, and 113 days postinoculation. Based on low *X. fastidiosa* concentrations in stem tissues, *M. rotundifolia*, *V. arizonica/candicans*, *V. arizonica/girdiana*, *V. candicans*, *V. girdiana*, *V. nesbittiana*, *V. shuttleworthii*, and 9621–67 were resistant to PD. Conversely, *V. vinifera* var. Chardonnay, *V. aestivalis*, 9621-94, and *V. champinii* had very high *X. fastidiosa* concentrations in xylem tissues, thus proving to be susceptible. However, the bacterial colonization dynamics in the stems among the genotypes differed significantly from those in leaves. As a summary of the trial, *M. rotundifolia* was the only species in which *X. fastidiosa* was detected in both stem and leaf tissues, albeit to a lesser extent. As the greenhouse phenotyping protocol was continuously improved, Gambetta et al. (2007) did not observe correlations between LS symptoms and localized bacterial concentrations in the susceptible variety Chardonnay, casting doubt on a pathogenesis mechanism based on vascular occlusions. The study, which was based on the use of a sensitive qPCR method to assess bacterial populations in leaf and vein tissues, concluded that there was no association between the presence of high *X. fastidiosa* populations and symptoms, but the involvement of a systemic plant response in the pathogenesis was invoked. Under controlled conditions, Saunders et al. (2023) evaluated 21 grape varieties using two *X. fastidiosa* subsp. *fastidiosa* strains, the moderately virulent Stag’s Leap and the more aggressive N-001. Varieties Tampa, B43-17, and 8909-08, previously reported as tolerant/resistant, consistently exhibited mild symptoms, even when inoculated with the strain N-001. USDA breeding lines, several table grapes, and the winegrape Cabernet Sauvignon were moderately to highly susceptible, showing great variability in disease symptoms and relatively high bacterial concentrations. The genotype IAC-572, a *V. caribaea* hybrid rootstock, manifested very few PD symptoms with both strains, albeit harboring high bacterial populations when inoculated with isolate N-001, thus representing a promising source of resistance.

In a completely different geoclimatic environment, Martínez et al. (2023) assessed PD susceptibility of major European *V. vinifera* varieties and commercial rootstocks (R110, P1103, 196–17 Cl, 41B-MGt, Ru140), using local Spanish isolates of *X. fastidiosa* subsp. *fastidiosa* ST1 (XYL 2055/17 and XYL 2177/18). Across multi-year field (2019–2020) and greenhouse (2021) trials in Mallorca, the authors found that the isolate XYL 2055/17 was consistently the most aggressive. Although all tested *V. vinifera* varieties, mainly Spanish and widely cultivated, showed symptoms, they could be distinguished in a wide range of susceptibility. All rootstocks presented very low susceptibility to the pathogen but, when varieties were grafted on them, the disease progression in the shoots of the scions appeared not significantly altered. On the contrary, the environmental conditions had a major impact: symptoms progressed faster and more severely in the field than in the greenhouse. Recently, Kahn et al. (2023) tracked the PD progression in three artificially inoculated mature plants of Cabernet Sauvignon, Merlot, and Chardonnay, grafted on 101–14 rootstock, planted in two different fields in Napa Valley. Trials encompassed more than 2 years, and plants were exposed to biotic (other pests and pathogens) and abiotic (nutrient deficiencies) factors otherwise not occur in a greenhouse. Contrary to the prevailing PD paradigm that LS is the first observed symptom, the authors observed a high overwinter recovery unexpectedly and found that the shriveling of berries was the most reliable symptom, often appearing only three months after inoculation and preceding the common LS, particularly in Cabernet Sauvignon. The bacterial spatial pattern also changes over time: in the first year, the presence of both bacterial cells and symptoms was concentrated close to the inoculation site, but they began to be homogeneously distributed across the plant by years 2 and 3. Overall, this study emphasizes the need for *in situ* trials to evaluate the behavior (tolerance or susceptibility) of grape varieties to the bacterium and the value of symptom-based ranking for early field detection, when qPCR failed.

## 7 Genetic architecture of Pierce’s disease resistance

### 7.1 Geographic patterns of resistance sources

As soon as the epidemiology of the disease was better understood, it became apparent that a geographic pattern of PD severity, related to climatic conditions, existed and overlapped with the distribution of resistant grape species. A warmer climate would favor high vector populations and longer growing seasons, both factors contributing to the high incidence of the disease. Grape species originating from the southeastern USA with high natural PD pressure, like Florida, the coastal areas of Texas and North Carolina, such as *V. rotundifolia*, and southern Arizona and New Mexico, such as *V. arizonica*, evolved resistance through a long process of coexistence with the pathogen. Ruel and Walker (2006) estimated the resistance of five native species by quantitatively assessing bacterial concentration in the stem using ELISA and scoring symptoms in greenhouse conditions. *V. labrusca* (five accessions), originating from the PD-free area of the Northeastern USA, were as susceptible as the three *V. vinifera* varieties, Chardonnay, Chenin Blanc, and DVIT1800 (a wild selection from Afghanistan), employed as positive controls. Accessions of *V. rotundifolia* (30) and *V. arizonica* (two) were highly resistant, while native Californian species, *V. californica* (two) and *V. girdiana* (seven), had an intermediate behavior. In particular, *V. girdiana* accessions showed the widest range of responses: three plants collected in PD-severe areas were as resistant as *V. rotundifolia* and *V. arizonica*, while the remaining ones, from inland desert areas where PD is not present, were susceptible, suggesting that resistance cannot be ascribed to drought because of the geographical origin of the susceptible accessions. Buzombo et al. (2006) moved to vineyards in the Gulf of Texas, a PD hot zone, to analyze the crop performances of three hybrids: Blanc du Bois, an interspecific hybrid of *V. aestivalis* with parentage including Cardinal, Golden Muscat, and Pixiola; Black Spanish, an interspecific hybrid of *V. aestivalis*, *V. cinerea*, and *V. vinifera*; and Cynthiana (synonym Norton), a natural hybrid of *V. aestivalis* and *V. labrusca*. Over a 10-year period, growers reported a stable crop yield for Blanc du Bois and a dramatic decrease for Cynthiana, which was the most affected. The yield data were further confirmed in summer 2003, when also Black Spanish, which was introduced into the study, also performed similarly to Blanc du Bois and much better than Cynthiana. The lower bacterial population levels detected by ELISA in Blanc du Bois and Black Spanish led the authors to propose that the two resistant varieties confined the bacterium, which is subsequently eliminated by pruning operations; whereas this compartmentalization did not occur in Cynthiana, as the bacterium was not eliminated by pruning operations and overwintered in the stem, reaching its highest population size in August because of new insect inoculations.

### 7.2 Identification and characterization of the *PdR1* locus

The 9,621 progenies of D8909-15 × F8909–17 developed by Walker and collegues (Doucleff et al., 2004) were further investigated to identify and map the first PD resistance gene, named Pierce’s disease resistance 1 (*PdR1*). Bacterial measurements in stem tissues by ELISA allowed the localization of a large quantitative trait locus (QTL) on the genetic linkage group 14 (LG 14) of the male parent F8909-17. The other two evaluation methods, CMI and LS, confirmed this QTL localization, although with a broad confidence interval. No major QTL was identified in the female parent (D8909-15), which had a *V. arizonica* Baja California parentage (b42-26), whose resistance was likely based on the effects of multiple minor genes. The mapping of the *PdR1* locus laid the foundation for the implementation of a more precise breeding program aimed at introgressing the resistance alleles into *V. vinifera* varieties. However, due to the high heterozygosity of grape varieties, several generations of backcrossing with high-quality *V. vinifera* varieties would have been required. In the meantime, the discovery of molecular markers associated with *PdR1* would help to remove susceptible genotypes from future breeding populations, thus accelerating the development of PD-resistant germplasm.

A refined mapping of the *PdR1* locus was obtained by Riaz et al. (2006) using a different set of progeny plants of the “9621” population, through a combination of DNA-derived SSR, expressed sequence tags (EST)-derived SSR, EST-STS, and EST-RFLP markers. Particularly, 181 genotypes of the 9621 population were phenotyped by ELISA measurements and the CMI index as described in Krivanek et al. (2005b, 2006). Although intended for *V. vinifera* species, the genomic DNA-derived SSRs were found to be highly reproducible and polymorphic also in the non-*V. vinifera* species among the 9,621 plants, allowing their use in interspecific mapping projects. Moreover, the adoption and development of new EST-derived SSR markers enhanced the map information, as they were derived from transcribed regions possibly responsible for traits of interest, unlike the anonymous genomic DNA-derived SSRs. This extended genomic map provided a more detailed map of the *PdR1* resistance locus (201 kb) in LG 14 of the F8909–17 male parent (*V. rupestris* “A. de Serres” × b43-17), and a set of additional markers useful for future MAS breeding activities. Complementing the resulting map information, the transcriptome profile generated by the *X. fastidiosa–*grape interaction was studied in susceptible and resistant genotypes. Differentially expressed transcripts were identified by subtractive hybridization of xylem cDNA libraries of the moderately tolerant *V. vinifera* variety Zinfandel vs. the tolerant Blanc du Bois (Vasanthaiah et al., 2007). The presence of genes expressed only in the tolerant genotype could suggest a possible adaptation to pathogen infection. A more detailed transcriptomic study was carried out by Lin et al. (2007), who performed a comparative EST analysis of the resistant (9621-67) and highly susceptible (9621-94) sibling genotypes of the 9621 population, using stems, leaves, and shoot tissues collected at different times after inoculation. The authors observed that, upon *X. fastidiosa* infection, the stem library from resistant genotypes was enriched in β-1,3-glucanase and 14-kDa proline-rich protein, as well as primary cell-wall-modifying proteins, such as xyloglucan endotransglycosylase (XET), endoxyloglucan transferase (EXT), and enzymes related to energy and defense. In contrast, stem libraries from the susceptible variety were enriched with transcripts encoding Pathogenesis-related (PR) proteins and osmotin-like protein TPM-1, glucan 1,3-β-glucosidase, and proteolytic pathway proteins such as aspartic protease, the beta 7 proteasome subunit, and the 20S proteasome beta subunit (PBG1). Only the shoot library from infected susceptible genotypes had ESTs (a chitinase-like protein, a nonspecific lipid transfer protein precursor (LTP), and an F-box/LRR-repeat protein-20) that responded to the pathogen. Therefore, the comparisons between stem libraries from resistant and susceptible plants produced the most informative EST expression patterns: the enriched expression of ESTs related to cell-wall-modifying enzymes in the resistant genotype was indicative of an activity aimed at reinforcing physical barriers to limit pathogen invasion.

In addition to the 9621 population, the F8909–17 full sibling F8909-08 was crossed with the susceptible *V. vinifera* F2–7 to give the 04190 population (Riaz et al., 2008). Both populations, phenotyped for the response to *X. fastidiosa* inoculation in greenhouse conditions, showed a 1:1 segregation ratio of PD resistance, indicating that both parents (F8909–17 and F8909-08) were heterozygous for resistance, which is controlled by a single dominant locus or gene. The refined genome maps of LG 14 of these two populations revealed that the two siblings inherited different chromatids from b43-17. These findings indicate that the F8909–17 and F8909–08 lines inherited one of the two alleles of the same gene, which were named *PdR1a* and *PdR1b*, respectively. Another accession, *V. arizonica* b40-14, collected in Chihuahua (Mexico), was also found homozygous for PD resistance, and its associated locus, *PdR1c*, was mapped to the same genomic region, supporting the importance of LG 14 (Walker and Riaz, 2009; Walker et al., 2014). The availability of a phased diploid genome for this accession (b40-14) enabled single haplotype-resolved analyses and allele-specific expression approaches that narrowed the locus to a small set of strong candidates, notably four extracellular receptor genes of the RLP/RLK-type, proposed to underlie the *PdR1*-mediated resistance (Paineau et al., 2025).

Starting from 4,360 seedlings and exploiting a MAS approach, Walker et al. (2014) backcrossed resistant selections with different elite winegrape varieties, achieving a very high *V. vinifera* content (e.g., 94% and 97%), and halving the number of seedlings planted in the field for PD evaluation from approximately 4,360 to 2,000.

The dawn of genome mapping by molecular markers prompted their use in muscadines to profile varieties and hybrids (Riaz et al., 2008), due to their multiple resistance to powdery mildew, root-knot and dagger nematodes, phylloxera, and PD. Taking advantage of SSR markers developed for *V. vinifera* and *V. riparia*, it was possible to authenticate the identity of *M. rotundifolia* and its hybrids from two available repository collections and to identify 88 SSR-derived alleles that were unique to muscadine varieties. Using the F1 population derived from *M. rotundifolia* Fry × Trayshed, Riaz et al. (2012) built a consensus genetic map of *M. rotundifolia* based on SSR markers that showed a high level of synteny with *V. vinifera* across the 19 LGs; a finding that indicated that, despite their evolutionary divergence, the two genomes share a largely conserved structure. However, they also have skewed regions that could explain the resilience of muscadine to a broad spectrum of pathogens, including *X. fastidiosa*, which could be introgressed into cultivated grapevine through breeding programs.

### 7.3 Evolution and diversification of PD resistance in wild *Vitis* species

Relying on the single *PdR1* locus makes resistance durability risky, particularly when tested in different environments. To enlarge the genetic base of resistance, Riaz et al. (2018) characterized a set of resistant species using chloroplast and SSR markers to determine their genetic diversity and uniqueness. Among *V. candicans*, *cinerea*, and *girdiana* accessions and their interspecific hybrids originating from the southwestern USA and Mexico, nine had a major QTL mapped to the *PdR1* locus on chromosome 14, while three had their resistance loci located outside the *PdR1* region of the genome, thus expanding the repertoire of loci for breeding. To identify these loci, each resistant accession was crossed with a susceptible *V. vinifera* genotype, generating F1 progenies, which were subsequently phenotyped for the response to PD infection and subjected to QTL analysis. Interestingly, no geographical correlation was observed, since the nine accessions were collected from the southwestern USA and Mexico. The identification of the same locus in geographically distinct accessions led the authors to hypothesize that PD resistance may rely on broad-spectrum defense mechanisms and not on direct interaction with the pathogen. These mechanisms likely include lignin accumulation, antimicrobial production, induction of PR proteins, and a hypersensitive response, which is typical of a nonhost response. A global summary of the breeding program started at UC Davis in 1990 using the original *V. rupestris* × *M. rotundifolia* crosses made by Olmo was reported by Dry et al. (2019).

The development of a set of 20 SSR markers allowed Riaz et al. (2020) to carry out the genetic analysis of 266 *Vitis* accessions reported as PD resistant, by evaluating results from 19 trials performed from 2011 to 2018. A standardized comparison among the trials was ensured by the adoption of the same set of control genotypes and common disease evaluation parameters. Bayesian clustering of the allele frequencies of SSR markers showed that a phylogeographic barrier, consisting of the Sierra Madre Oriental mountains, distinguished accessions in two genetically distinct groups according to an east–west division, with the state of Texas being a potential hybridization zone. Grape accessions from the southwestern USA and Mexico, which have a drier climate, are genetically separated into three subclusters based on different habitats: *V. arizonica*/*V. treleasei* (a glabrous form of *V. arizonica*), *V. arizonica*/*V. girdiana*, and *V. arizonica*/*V. rupestris*. The second group contains *Vitis* species native to the wetter climate of the southeastern USA, Eastern Mexico, and Costa Rica. It is divided into four subclusters: *V. cinerea*/*V. berlandieri*, *V. candicans*/*V. monticola*, *V. aestivalis*/*V. labrusca*, and *V. cinerea*/*tiliifolia*. Along this east-west division, grape accessions from the southwestern area had stronger resistance to PD, with lower bacterial populations and levels of cane lignification and LS/LL than species from the southeastern USA, all having higher levels of these three traits. Thereby, southeastern accessions could be more tolerant and resilient to infection because of the more humid climate conditions, which attenuate symptoms, although they do not control the bacterial populations. *V. arizonica* and *V. girdiana* showed strong and moderate resistance, respectively, while *V. cinerea* accessions from Mexico and *V. candicans* were the only species displaying strong resistance among accessions from the southeastern USA.

Greenhouse trials carried out from 2011 to 2020 (Krivanek et al., 2005b; Riaz et al., 2020) allowed the evaluation of the evolutionary patterns and introgression events of six wild *Vitis* species native to the southwestern USA to find evidence of biotic and abiotic adaptation (Morales-Cruz et al., 2021). Historical introgression was analyzed by generating a *V. arizonica* reference genome and producing a dataset of *ca*. 20 million SNPs by resequencing 130 accessions of *V. girdiana*, *V. riparia*, *V. candicans*, *V. berlandieri*, *V. monticola*, and *V. arizonica*. Putatively introgressed regions (pIRs) were enriched in SNPs located in regions containing genes involved in disease resistance and climate adaptation. Widespread introgression among the species and the exchange of genomic regions have been maintained because they provide adaptive benefits against both abiotic (climate) and biotic (PD) stressors. A subset (3.6 million) of the produced SNPs was used by Aguirre-Liguori et al. (2022) to assess which of the five *Vitis* species (*V. arizonica*, *V. mustangensis* synonym *V. candicans*, *V. berlandieri*, *V. girdiana*, and *V. riparia*) analyzed by Morales-Cruz et al. (2021) have alleles that can help mitigate the effects of climate change on viticulture. The integration of data revealed that *V. mustangensis* has potential alleles better suited to withstand future climate change. Riaz et al. (2023) investigated the genetic basis of PD by analyzing grape germplasm collected from the southwestern USA and Mexico, crossing resistant wild species with susceptible genotypes and enabling the segregation of resistance traits in the progeny. Using molecular markers and QTL analysis, they identified several genomic regions significantly associated with reduced bacterial colonization and milder disease symptoms.

A further study led to the description of a super-pangenome of North American wild grapes by assembling the genomes of nine *Vitis* species (*V. acerifolia*, *V. aestivalis*, *V. arizonica*, *V. berlandieri*, *V. girdiana*, *V. monticola*, *V. mustangensis*, *V. riparia*, and *V. rupestris*). Nucleotide genetic variants associated with PD were recapitulated and were found to be more numerous in the accessory genome compared to the core genome. These findings highlight that genetic regions associated with biotic stresses are disproportionately represented, particularly contributed by the variable component of the genome (Cochetel et al., 2023). Another pangenome was built by Huff et al. (2023), who assembled a long-read, chromosome-scale genome of the tolerant muscadine Carlos. Using this genomic resource, plus two other *V. rotundifolia* (Trayshed and Noble) and one *V. vinifera* genome, these authors assembled a *Vitis* pan-genome. A comparative genomic analysis revealed that muscadines, especially var. Carlos, present a greater number of resistance genes than *V. vinifera*, suggesting an improved ability of muscadines to counteract infections caused by *X. fastidiosa* and other pathogens. Moreover, RNA-seq on Carlos plants that were artificially inoculated and incubated for 4 weeks allowed the observation of more than 200 differentially expressed genes (DEGs), mainly associated with lipid catabolism, oxidation-reduction signaling, lignin-degrading processes, ABA biosynthesis, and plant defense response (like the kinase R gene class). When benchmarked with public RNA-seq data from infected *V. vinifera* plants, the Carlos transcriptome analysis revealed that most of the DEGs were uniquely expressed in muscadines, supporting an exclusive host response not found in common grapes. Agüero et al. (2022) extensively investigated the molecular basis of PD resistance in the highly resistant accession b43–17 through the *PdR1b* allele. By physically mapping the locus, they refined a narrow genomic interval containing five resistance gene analogs (from RGA14 to RGA18), with features typical of LRR receptor-like proteins (RLPs), and compared their sequences with those present in the *V. vinifera* reference genome (PN40024), highlighting major structural/regulatory differences that could affect gene function. Transgenic expression of RGA14 or RGA18 in susceptible Thompson Seedless, Chardonnay, and *V. rupestris* St George did not protect against *X. fastidiosa* infections, thus supporting their nonexclusive role in the resistance mechanisms.

An important advancement in the identification of candidate resistance genes in wild *Vitis* species was achieved by Morales-Cruz et al. (2023), who updated the reference genome of *V. arizonica* accession b40-14, homozygous for PD resistance, and resequenced 167 accessions sampled across the southwestern USA and northern Mexico. Exploiting these new genomic resources, SNPs associated with resistance were identified, revealing eight major peaks across five chromosomes. The most distinct signals were detected on chromosomes 14 and 15. Further confirming the role of the *PdR1* locus in the resistance mechanism, a kmer and copy number variation (CNV) analysis revealed that four of the eight peaks were strongly associated with the bacterial population levels in the analyzed accessions. A total of 124 genes were identified in genomic regions underlying these eight peaks, mainly implicated in the plant immune response. Among them, leucine-rich receptor-like proteins (LRR-RLP) and LRR-like kinases (LRR-RLK), as well as genes related to phytohormone-mediated defense mechanisms and antimicrobial activity, were found. Notably, two out of four candidate LRR-RLKs and LRR-RLPs (R-genes) were upregulated in infected resistant siblings, and none of them had a high degree of homology to those that failed to induce transgenic resistance by Agüero et al. (2022), suggesting that a novel resistance mechanism could be involved. In summary, kmer and SNP analysis depicted a scenario in which multiple genes and functions may contribute to PD resistance, with a predominant role played by disease-resistant (R) genes. The study allowed the selection of 99 resistance (R)-associated and susceptible (S)- associated kmers, which were used to investigate the distribution of the resistance markers in North American wild *Vitis* species. The additional analysis of 105 accessions showed an enrichment of R-kmers within a subcluster of resistant *V. arizonica* and *V. girdiana* accessions. In this latter species, five R-kmers mapped to chromosome 15, thus confirming the involvement of the non-*PdR1* region in the resistance. By contrast, R-kmers were mapped at a lower frequency on resistant accessions of *V. riparia*, *V. monticola*, *V. berlandieri* and *V. mustangensis* (synonym *V. candicans*), indicating that these species could have independently evolved alternative mechanism (s) of resistance which were not introgressed from *V. arizonica* or *V. girdiana*.

The search for additional unique sources of resistance was also undertaken by Huerta-Acosta et al. (2023) with the aim of widening the genetic background of resistance. These authors started from a natural hybrid of *V. arizonica* × *V. monticola* (accession b46-43) from Texas, which manifested fewer symptoms and a lower bacterial population than b43-17, thus suggesting a different genetic background. Segregation analysis performed on the F1 population obtained by crossing b46–43 seedlings with susceptible *V. vinifera* indicated that resistance had a homozygously dominant nature. Genetic mapping and QTL analysis revealed that no minor loci were identified in chromosomes other than 14. The mapping of the b46–43 resistance locus in the same *PdR1* genomic region identified in b43–17 suggests that this locus may have a common evolutionary spread among wild *Vitis* species through introgression events. A total of 323 grapevine accessions located at the Fondazione Edmund Mach (FEM) facilities were characterized using 29 SSR markers to quantify genetic diversity and population structure, and screened for the well-established 15 resistance loci (R-loci) of relevant grapevine diseases (downy mildew, powdery mildew, black rot, phylloxera, and PD). These accessions, which originated from seeds collected from open-pollinated female vines and are present in the University of California, Davis (USA) germplasm collection, were categorized into six North American species: 50 V*. aestivalis*, 61 V*. arizonica*, 90 V*. cinerea*, 98 V*. monticola*, 12 V*. mustangensis*, eight *V. rotundifolia*, and four unknown species. The *PdR1* locus was identified only in five genotypes (1.5%), two of which were classified as *V. arizonica* and the remaining three as *V. aestivalis* var. *aestivalis*, representing a valuable resistance donor (de Oliveira et al., 2025), further supporting the hypothesis that additional non-*PdR1* genomic regions may contribute to PD resistance in wild *Vitis* species.

### 7.4 The discovery of diverse resistance mechanisms and *PdR1* multiple haplotypes

Initial description of the gene organization of the *PdR1* locus did not provide clear indications of the mechanism(s) underlying the resistance. In trials using a wild *X. fastidiosa* strain and a prtA^−^ mutant lacking biofilm formation and hypervirulent in the susceptible variety Thompson Seedless, Huerta-Acosta and co-authors (2026) found that the hybrid U0505-01 (*V. vinifera × V. arizonica*) was resistant to both the planktonic and biofilm forms of the bacterium, while tolerant and susceptible accessions U0505–35 and U0505-22 were resistant only to the planktonic form but remained vulnerable to the biofilm form (Huerta-Acosta et al., 2026). These findings reveal the existence of multiple and complex mechanisms of plant response, whose outcome depends on the interactions between the bacterium and the genetic background of the grapevine accession. Complexity is also revealed by the discovery of different *PdR1* haplotypes (*PdR1a–f*) at the same locus (Massonnet et al., 2026) in *V. arizonica* and its hybrids. Integrating data from newly assembled and existing genomes of PD-resistant accessions, these authors found significant differences among the intergenic regions of the *PdR1* and alternative haplotypes, as well as diverse gene organizations, which led to the identification of LRR-RLP genes potentially involved in PD resistance. Resistance mechanisms distinct from the previously characterized *PdR1* locus on chromosome 14 were further identified in the b42–26 accession (*V. arizonica/girdiana*) (Riaz et al., 2026). Genetic mapping led to the discovery of a *q-PdR2* locus and suggested that b42–46 resistance relies on multiple genetic loci operating additively, as shown by the identification of three QTLs on chromosomes 8, 14, and 17. The possibility of stacking *PdR1* and *q-PdR2* loci would expand the genetic background favoring a stronger and more durable resistance (Riaz et al., 2026).

**Table 1** summarizes the genetics of resistance to *X. fastidiosa* subspecies *fastidiosa* in *Vitis*. The *PdR1* major resistance locus and its haplotypes a–f, identified on chromosome 14 of diverse genotypes of *V. arizonica* and interspecific hybrids (*V. arizonica/candicans*), are indicated. Additional loci, including unknown and *qPdR2* loci, located on chromosome 14 or other chromosomes, are reported. The table also describes the genes, mainly encoding RLP or RLK proteins, identified in these loci.

**Table 1:** Major resistance loci and candidate genes associated with PD resistance in grapevine, as well as the indication of the genotypes in which they were identified.

Resistance locus/gene	Genetic source	Chromosome	Proposed role in PD resistance
*PdR1*	*Vitis arizonica* (b43-17, b40–14 and related accessions)	Chr. 14	Major resistance locus (QTL)
*PdR1a*	F8909-17 (*Vitis arizonica/candicans*)	Chr. 14	*PdR1* allele
*PdR1b*	F8909-08 (*Vitis arizonica/candicans*)	Chr. 14	*PdR1* allele
*RGA14-RGA18*	*PdR1b* interval (b43-17)	Chr. 14	Resistance gene analogs (RGAs) LRR receptor-like proteins (RLPs)
				*PdR1c*	*Vitis arizonica* b40-14 Chihuahua (Mexico)	Chr. 14	*PdR1* allele; it contains four extracellular receptor genes (RLP/RLK-type)
*PdR1d–f*	*Vitis arizonica* accessions and hybrids (b46-43, b42-26)	Chr. 14	Alternative *PdR1* alleles contain LRR-RLP genes				
*Unknown*	*Vitis candicans*, *cinerea*, and *girdiana* accessions	Outside *PdR1* region	Collected from the southwestern US and Mexico area
*q-PdR2*	*Vitis arizonica/girdiana* b42-26	Chrs. 8, 14, 17	Quantitative resistance locus
*Unknown*	*Vitis arizonica* accessions	Chrs. 14 (outside *PdR1* region) and 15	It contains receptor-like proteins (R genes): LRR-RLPs and LRR-RLKs

The well-characterized *PdR1* locus and its alleles (*PdR1a–f*) on chromosome 14 of *V. arizonica* remain the primary source of resistance used in breeding programs. Additional resistance-associated loci (unknown, *qPDR2*) lying on diverse chromosomes of *V. candicans*, *V. cinerea* and *girdiana* and in geographically distinct *V. arizonica* accessions are reported, supporting a more complex and polygenic architecture of resistance.

### 7.5 *PdR1*-targeted breeding programs

Attempts to introgress PD resistance in table and wine grapes essentially exploited the well-characterized *PdR1* locus. Two resistant genotypes, D8909-15 (*V. rupestris* × *V. arizonica/girdiana* b42-26) and F8909-08 (*V. rupestris* × *V. arizonica/candicans* b43-17), were hybridized with different *V. vinifera* seedless varieties to produce F1 populations that were screened for *X. fastidiosa* resistance by symptom observation and bacterial population quantification by ELISA. Cluster, berry, and seed weights of the resistant and susceptible progenies from the three populations did not differ, indicating that the introgression of resistance genes does not affect fruit quality (Ramming et al., 2009). Seedlings carrying resistance genes from the D8909-15 × *V. vinifera* and F8909-08 × *V. vinifera* families were selected by MAS and planted in California, Texas, and Alabama for field evaluations under natural disease pressure (Walker et al., 2011). The b43-17-based selection F8909–8 was repeatedly backcrossed with *V. vinifera* to eliminate unwanted traits (Riaz et al., 2009). Three PD-resistant selections with an 87.5% *V. vinifera* background, namely U0501-12, U0501-10, and U0501-01, were tested at the Chilton Research and Extension Center, Clinton, Alabama, under high PD pressure. A 2-year assessment demonstrated that selections remained PD-free while maintaining high cropping potential and plant vigor (Coneva, 2016; Svyantek et al., 2016).

The formal release and the commercialization of the five PD-resistant winegrape varieties, Camminare Noir (US Patent No: PP32929), Paseante Noir (US Patent No. PP33039), Errante Noir (US Patent No. PP32999), Ambulo Blanc (US Patent No. PP32949), and Caminante Blanc (US Patent No. PP33015), are the endpoint of more than two decades of breeding effort carried out at UC Davis facilities and focused on the introgression of the *PdR1* locus characterized in the *V. arizonica* accession. These patented selections retain a predominantly *V. vinifera* genetic backgrounds (*ca*. 94%–97% vinifera) and were validated in greenhouse and multisite field trials in PD “hot spots” (e.g., Napa River plantings, Temecula, Sonoma), pilot/commercial-scale winemaking, and sensory tastings to assess whether wine bouquet and quality remain competitive with traditional *V. vinifera* standards, alongside assessment of the resistance (Almeida et al., 2019). More recently, the increasing availability of diploid genome references for wild *Vitis* species, combined with resistance-haplotype phasing, genome-wide association, and transcriptomic analyses, has significantly expanded the ability to elucidate the genetic architecture of PD resistance. This progress is generating candidate resistance genes that will support precision breeding, rational gene stacking, and genome editing in grapevine improvement programs. However, the major criticism concerns the localization of multiple PD resistance traits to the same region on chromosome 14 in *V. arizonica*. As distinct *PdR1* alleles are tightly linked, their stacking by conventional recombination is difficult. Therefore, targeted knock-in strategies to stack *PdR1* variants within the same locus could represent a promising strategy to facilitate their introgression. Although a *PdR1* breakdown has not yet been documented, the large-scale deployment of a single-locus resistance could select for pathogen adaptation, especially given *X. fastidiosa’s* capacity for genetic exchange *via* homologous recombination (Paineau et al., 2025).

## 8 Bacterial pathogenesis and host–plant interactions

From the 2000s onward, with the advent of molecular and omics technologies, research efforts increasingly focused on dissecting bacterial virulence mechanisms and the molecular basis of host–pathogen interactions. Lu et al. (2003) found that physiological and biochemical changes in grapevines occurred during the infection process by applying *X. fastidiosa* culture filtrates to wounded leaves of susceptible and resistant varieties. Severe symptoms developed in susceptible varieties, while resistant ones exhibited no or very mild symptoms, suggesting that the infection process alters transcript expression more significantly in susceptible genotypes than in resistant ones. Cheng et al. (2009) hypothesized that differences in the sap and cell-wall composition of resistant (9621-67) and susceptible (9621-94) selections could influence pathogenesis mechanisms (i.e., *X. fastidiosa* growth, biofilm formation, and cellular aggregation). In addition, these authors hypothesized that the sap composition changes during the infection process, since CWDEs determine the release of cell wall components. Their studies demonstrated that media amended with sap from PD-susceptible varieties support greater *in vitro* growth and biofilm formation than media containing sap from resistant grapes. Most of the cell wall constituents, when amended to the medium, increased bacterial growth at the late stage of *in vitro* culture, indicating that the degradation and use of host cell-wall components may be key mechanisms of *X. fastidiosa* pathogenicity upon infection. Along the same line of research, Zaini et al. (2009) found that adding xylem sap from the susceptible species *V. riparia* to the culture medium increased growth and biofilm formation compared with the medium alone. Shi et al. (2013, 2014) found better growth of *X. fastidiosa* cells in pure xylem fluid of PD-susceptible grapevines (*V. vinifera* and *V. labrusca*) than in PD-resistant grapevines (*V. champinii* and *V. smalliana*), thus confirming the previous observations of Zaini et al. (2009). Additionally, DNA microarray analysis confirmed that key genes, mainly involved in virulence, motility, and attachment to surfaces, were more highly expressed in cells grown in the susceptible sap environment, while their expression was downregulated in bacterial cells recovered from resistant grapevine saps. Overall, these studies suggested that the chemical composition of xylem fluid plays a crucial role in regulating bacterial virulence and growth and could help to explain the resistance observed in certain grape species. Finally, Hao et al. (2016) compared biofilm formation and growth density of *X. fastidiosa* strain Temecula 1 in different sap-renewal cultures from PD-susceptible and PD-resistant species. Fresh sap from the susceptible Chardonnay and New York plants, as well as from *V. riparia*, supported much higher biofilm formation and higher numbers of viable cells than saps from the resistant species *V. aestivalis*, *V. champinii*, and *V. arizonica*, where growth and biofilm formation were strongly suppressed. These results were also confirmed in a microfluidic chamber system that mimics the xylem vessel environment. In this system, the bacterium formed numerous small star-shaped aggregates in *V. champinii*, *V. arizonica*, and *V. cinerea* saps rather than a mature and robust biofilm, as occurs in *V. vinifera* saps. An important exception was *V. mustangensis*; although classified as PD-resistant, its sap supported a dense bacterial cell lawn that still lacked the characteristic 3D mature biofilm structures. This observation suggested that resistance could involve mechanisms beyond the inhibition of biofilm production.

## 9 Influence of grapevine genotype on vector feeding behavior

In the late 1980s, Alexander Purcell investigated whether the blue-green sharpshooter vector, *Graphocephala atropunctata*, had a feeding preference for some European grape varieties cultivated in California that could explain their different susceptibility to PD. His studies demonstrated that the insect feeding preference is not directly correlated with resistance but rather is influenced by other factors, such as the age of plant tissues and the timing of infection during the growing season (Purcell, 2025). Fritschi et al. (2007b) studied the role of grape genotypes in the transmission efficiency of *Homalodisca vitripennis* (*Hemiptera*: *Cicadellidae*) under controlled conditions. In choice trials, independently of prior acclimatization to one of the four tested genotypes, the susceptible Chardonnay was always preferred by the vectors, while the tolerant *V. candicans* was the least preferred. The D8909–17 line and *V. girdiana* showed intermediate and less consistent levels of attractiveness.

Attempts to correlate the amino acid compositions of the xylem sap of the four genotypes with insect preference did not reveal significant relationships. However, insect mortality was threefold higher on *V. candicans* than on Chardonnay, leading the authors to conclude that grape genotypes may influence *H. vitripennis* behavior, consequently affecting *X. fastidiosa* transmission and PD epidemiology. Backus et al. (2021) further demonstrated by electropenetrography (EPG) that trichomes on leaves of the resistant *V. champinii* (*V. candicans* × *V. rupestris*) may constitute a temporary physical hindrance to the vector probing activity. Although the scraping of trichomes did not provide further clues about their effects on insects’ behavior, data showed that insects spent a significantly longer period of inoculation on Chardonnay than on *V. champinii*, regardless of the trichomes’ presence. Combining the present results with those of Fritschi et al. (2007b), the authors suggest that *V. champinii* xylem sap is accepted by *H. vitripennis*, although insects were constrained by trichomes. In contrast, insects can ingest Chardonnay sap for a longer period, albeit with more frequent salivation, likely because of the superior nutritional quality. Overall, these studies suggested that, in addition to breeding for resistance based on post-inoculation (bacterial infection and symptom development), the vector’s probing process and inoculation efficiency should also be considered as complementary tools for searching for PD resistance traits in breeding programs.

## 10 Engineering resistance: new frontiers in grapevine defense against *Xylella fastidiosa*

The first transgenic grapevine tolerant to PD was developed in 2005 by Agüero et al. (2005), who, leveraging the discovery that *X. fastidiosa* encodes cell wall-degrading PG, transformed susceptible Thompson Seedless and Chardonnay with a polygalacturonase-inhibiting protein (PGIP) from pear. The authors demonstrated that transgenic grapevines with increased PGIP activity were more tolerant to *X. fastidiosa* infections, showing reduced LS and lower bacterial populations in comparison with untransformed plants. PG inhibition was also the strategy pursued by Kirkpatrick et al. (2010) through the production of transgenic tobacco and grapevine expressing peptides or scFv antibodies specific for these bacterial enzymes. Although the results were preliminary, Dutt et al. (2007) grafted scions of Cabernet Sauvignon and Thompson Seedless onto transgenic Thompson Seedless used as rootstock, expressing the small Shiva-1 lytic peptide gene, which encodes a natural antibacterial compound similar to cecropin B and which inhibited the growth of bacterial cells in the xylem sap, thus providing PD resistance. In the same year, the release of the first *V. vinifera* genome variety, Pinot Noir, by two consortia (Jaillon et al., 2007; Velasco et al., 2007), as well as the genetic understanding of disease resistance (Dry, 2014), stimulated a series of studies to genetically improve elite *V. vinifera* varieties with a focus on disease resistance.

Transgenic lines of Thompson Seedless were also produced by Dandekar et al. (2012) by delivering an engineered protein chimera that combines a recognition domain (i.e., an elastase that specifically cleaves the conserved outer-membrane protein MopB) with a lysis domain (the antimicrobial peptide cecropin B), fused to signal peptides to target secretion into the xylem. Bioassays proved that the chimeric transgene suppressed bacterial growth and significantly reduced the development of symptoms. Moreover, proton-density NMR imaging of stem cross-sections of transformed grapes showed improved water density and no xylem clogging, further supporting this strategy as a promising alternative to conventional breeding. Some years later, the same authors (Dandekar et al., 2019) demonstrated, under field conditions, that protection against PD can be transferred from transgenic rootstocks that secrete either the chimeric antimicrobial protein (Dandekar et al., 2012) or the PGIP (Agüero et al., 2005) to nontransgenic, susceptible scions. Over a 7-year field trial, *X. fastidiosa*-inoculated transgenic lines showed a reduction in mortality (approximately 30%–95%), lower bacterial populations, milder symptoms, and improved vegetative vigor compared to the untransformed controls. A further transgenic strategy to mitigate PD in grapevine is reported in Lindow et al. (2014). The authors reprogrammed grapes var. Freedom (*Vitis champinii* × [*V. solonis* × *V. othello*]) to induce the production of the bacterial quorum-sensing diffusible signal factor (DSF) by transgenic expression of the synthase gene *rpfF*. The rationale of the strategy is that high concentrations of DSF in plant tissues would induce a shift of *X. fastidiosa* behavior toward a more adhesive and less diffusible lifestyle, thus limiting systemic colonization. In both greenhouse and field experiments with glassy-winged sharpshooter, transformed grapes exhibited markedly reduced disease severity and fewer symptomatic leaves and shoots, consistent with a strong reduction in the longitudinal and lateral bacterial movement along the xylem. Li et al. (2015) engineered *V. vinifera* variety Thompson Seedless and the hybrid Freedom with an antimicrobial lytic peptide, LIMA-A, to control PD infections. Transgenic lines remained *X. fastidiosa*-free in greenhouse conditions for 10 years, even after multiple artificial inoculations. However, when tested in the field, selected plants, including a transgenic rootstock, succumbed to PD over a 7-year trial. More recently, Agüero et al. (2022) introduced, via *Agrobacterium tumefaciens*, into susceptible grapes (Chardonnay, Thompson Seedless, and St. George) *RGA14* and *RGA18* genes from *V. arizonica*, whose gene structures differ from those of their homologs in *V. vinifera*. At 12 weeks postinoculation, a partial reduction in bacterial loads and milder symptoms were observed in some Chardonnay and St. George transgenic lines, as well as a reduced expression of pectin lyase and β-1,3-glucanase, suggesting that LRR-RLP proteins could work with coreceptors to trigger a stronger defense response. Leveraging the ability of the *X. fastidiosa* lipopolysaccharide (LPS) to elicit a plant immune response, Castro et al. (2023) reported that susceptible Cabernet Sauvignon, primed with the LPS, had less tylose development, lower bacterial loads, and reduced disease severity. Moreover, primed vines had a higher number of DEGs after bacterial inoculation than naïve (not primed) inoculated plants. A cationic peroxidase, VviCP1, upregulated in primed vines, mitigated disease symptoms when used to transform susceptible Thompson Seedless grapes.

## 11 Advances in the proteomic field

Preliminary efforts to analyze the proteome of xylem tissues of PD-resistant and susceptible grapes were conducted by Basha et al. (2003), who found different protein profiles of muscadines and bunch genotypes. These studies paved the way for Katam and Basha (2009), who characterized the xylem proteomes of tolerant *V. rotundifolia*, the Florida hybrid variety Suwannee, and the susceptible *V. vinifera* Pinot noir. The highest number of proteins was found in muscadines, followed by the hybrid and Pinot noir, with eight polypeptides uniquely expressed in *V. rotundifolia*, which were proposed to be associated with the tolerant phenotype. Further characterization of the xylem sap proteins from the aforementioned genotypes was performed by Basha et al. (2010), who identified a group of polypeptides abundant in *V. rotundifolia*, moderately present in Suwannee, and totally absent in Pinot noir. Liquid chromatography/mass spectrometry analysis (LC/MS) disclosed similarities between these proteins and plant defense β-1,3-glucanase and peroxidase enzymes. Yang et al. (2011) expanded the previous proteomic studies, increasing the protein resolution by employing MS and highlighting that differential expression of proteins depends on plant genotype, tissue phenological stage, and level of infection. Among the most expressed *X. fastidiosa-*induced proteins identified in both genotypes, there was a thaumatin-like protein, a PR protein homologous to osmotins. Further proteomic analyses of xylem sap and stem tissues revealed genotype-dependent protein expression patterns associated with PD resistance. Several defense-related proteins, including β-1,3-glucanase, COP9, and aspartyl protease nepenthesin precursor, were exclusively detected in PD-tolerant grapevines, whereas proteins associated with infection were identified in susceptible genotypes upon *X. fastidiosa* inoculation. In addition, three heat shock proteins constitutively expressed in resistant plants were suggested to contribute to PD resistance mechanisms (Katam and Lekan, 2015; Santos and Figueiredo, 2021). Chakraborty et al. (2016) also investigated the changes in the proteome composition of xylem sap upon *X. fastidiosa* infection by combining LC/MS with the CHURNER pipeline, which integrates conventional sequence-based homology with structural similarity searches and active-site comparisons to highlight potential functional relationships not obvious from simple sequence annotations. Using this combined strategy, they detected 91 proteins in the xylem sap from infected and noninfected plants; in particular, this approach allowed them to recognize increased levels of proteins linked to the defense response in infected plants, such as PR proteins (PR-1 and β-1,3-glucanases) alongside proteins involved in the cell-wall/vascular remodeling (chitinases, expansin, lipid-transfer protein), including a blue copper protein-like protein involved in lignin deposition. The stem cell-wall thickening of the infected grapevines, due to the deposition of dense material, was also observed through MRI imaging. The same protein families (PR proteins, chitinases, and peroxidases) were upregulated in infected symptomatic grapes analyzed by Sagawa et al. (2020), who compared the conventional gas-phase fractionation data-independent acquisition (DIA) libraries with a deep-learning-based approach that predicts the retention times and fragment ion intensities of proteins, generating *in silico* libraries. This pipeline proved to be more efficient than DIA, since it dramatically increased the resolution depth from 145 to 360 grape proteins and from 18 to 90 bacterial proteins, and expanded the detectable molecular-weight range, improving peptide coverage, particularly for low-abundance proteins.

## 12 Functional genomics studies of grapevine resistance and susceptibility

One of the first researchers to adopt a functional genomics approach to investigate grape resistance/susceptibility was Lin et al. (2007). He employed the subtractive suppression hybridization (SSH) EST technique to compare the transcriptomic profiles of highly resistant 9621–67 and susceptible 9621–94 selections derived from the F8909–17 population (*V. rupestris* × *V. arizonica/candicans*) in different plant tissues (leaf, stem, shoot) at 10 stages of PD development. Sequencing libraries obtained from stem tissues produced the most interesting EST expression patterns. The resistant accession had ESTs linked to primary cell wall modification and metabolic enzymes, and to some PR proteins such as β-1,3-glucanase, chitinases, and 14 kDa proline-rich protein. On the contrary, the susceptible grape expressed various PR protein classes, typically associated with infection or stress conditions. The same genomic technique was also applied by Vasanthaiah et al. (2007) to identify specific transcripts differentially expressed in susceptible and tolerant Florida bunch hybrid genotypes upon infection. A series of genes involved in metabolic pathways like energy modulation, translational control, and oxidative stress responses were greatly upregulated in tolerant genotypes exposed to the bacterium. The involvement of cytochrome P450 genes in the defense responses to *X. fastidiosa* (Lin et al., 2007) prompted further investigation into these enzymes, known to mediate the biosynthesis of primary and secondary compounds with antimicrobial activity (Cheng et al., 2010). In particular, the cytochrome P450 transcript (CYP736B) was found to be upregulated in the PD-resistant 9621–67 accession and downregulated in the susceptible 9621–94 genotype upon *X. fastidiosa* infection. The cloned CYP736B gene contains three candidate transcription start sites (TSS) and three polyadenylation sites (PolyA) whose frequency of use varied according to grape genotype, developmental stage, tissue, and treatment, thus regulating its expression.

Arnholdt-Schmitt et al. (2024) reanalyzed publicly available RNA-seq datasets from infected tolerant/resistant grapevines (*V. rotundifolia* var. Carlos and genotypes 094, 114, and 102) and from susceptible grapevines (var. Cabernet Sauvignon) to identify early, functional markers associated with multiresilience to *X. fastidiosa* and potentially other stresses. The study focused on mitochondrial respiration, highlighting the role of the alternative oxidase gene (AOX) as a rapid adaptive metabolic response when the cytochrome pathway (COX) is constrained. The authors propose that the ratio between COX/AOX transcripts and early Gleichschaltung (i.e., upregulation) of transcript levels of AOX genes represents a robust trait to discriminate between resistant and susceptible phenotypes and to support the development of markers associated with *X. fastidiosa* tolerance and multiple stress resilience. Following infection, particularly at early presymptomatic stages, Cabernet Sauvignon showed a strong and time-dependent reprogramming of the respiratory machinery, with a pronounced shift toward AOX-mediated respiration along with a decrease in the COX pathway. By contrast, resistant/tolerant genotypes displayed a more balanced COX/AOX relationship, comparable to that observed in mock controls, suggesting a form of “respiratory resilience” trait and a more stable redox homeostasis during the infection process. Moreover, in resistant genotypes, COX and AOX levels remained comparatively stable, consistent with tighter redox and stress buffering. Overall, this study supports the idea that resistance/tolerance is associated with a steadier capacity to adjust mitochondrial respiration and redox homeostasis during infection, whereas susceptibility is characterized by stronger respiratory stress responses. In parallel, the study further indicated that host genotypes may influence the activity of xylem-associated endophytic microbial communities, whose dynamics may contribute to grape tolerance. In Cabernet Sauvignon, the 16S rRNA signal, including the signal emitted by *X. fastidiosa*, exhibited a large fluctuation between asymptomatic and symptomatic stages, suggesting a change in xylem conditions over time that becomes progressively more permissive to bacterial activity. The role of long noncoding RNAs (lncRNAs) in grapevine responses to both biotic stresses, including *X. fastidiosa*, and abiotic stresses was investigated extensively by Cao et al. (2025) because they act as crucial gene expression regulators in plants. To induce their expression, grapevines were primed with lipopolysaccharide or sterile water for 4 h and then inoculated with *X. fastidiosa* suspension. RNA-seq of petioles collected at the inoculation site and 20 nodes above the inoculation site was performed to identify lncRNAs and mRNAs associated with the resistance. A computational weighted gene coexpression network analysis (WGCNA) allowed the identification of a small set of coexpression modules enriched in canonical immune-related pathways, including plant–pathogen interaction, hormone, and MAPK signaling. The authors proposed 11 pivotal lncRNA–mRNA regulatory pairs, notably a glycosyltransferase (GT2), an esterase/lipase (GELP), and an F-box protein as putative regulators of grape defense during early *X. fastidiosa* infection.

## 13 Influence of phenolic compounds on *Xylella fastidiosa* disease development in grape

Plant secondary metabolites, specifically phenolic compounds, are ubiquitous in plants and have been shown to possess antimicrobial properties. Phenolics produced by grapevines span a wide spectrum, from structural compounds that reinforce the cell walls, such as lignin (and its coumaric-acid-derived precursors) and tannins, to compounds associated with antibiotic activity (e.g., stilbenoids and flavonoids). One of the most relevant phenolics involved in grape defense against pathogens and/or abiotic stresses is resveratrol, which is a stilbenoid phytoalexin. Huang et al. (2007) *in silico* analyzed the gene sequences encoding stilbene synthases, the key enzymes that produce resveratrol, in both susceptible and resistant grape species to investigate whether amino acid variations might affect the structure of the protein involved in this plant defense mechanism. Wallis et al. (2013) observed that Chardonnay plants grafted onto Salt Creek or Freedom, as well as Cabernet Sauvignon grafted onto 101-14MG, 1103P, 420A, and Schwarzmann rootstocks, had reduced PD symptoms in greenhouse conditions over a 6-month experiment. The same authors observed that the milder symptoms in some combinations (Chardonnay/101-14MG) were associated with a greater phenolic concentration in the sap (caftaric acid, methyl salicylate, aprocyanidin trimer, and quinic acid), with these compounds being produced in response to the infection (Wallis and Chen, 2012). Although additional field studies are necessary for validation of these findings, these trials demonstrated that rootstock selection had effects on the tolerance of grafted varieties to PD. Indeed, total phenolic concentrations, particularly flavonoids, were approximately halved in leaves of resistant siblings (07744-094 and 07744-102) carrying the *PdR1c* locus, compared to those in susceptible lines (07744-007 and 07744-092), leading to the hypothesis that the consistently lower flavonoid levels in resistant vines may alter *X. fastidiosa* behavior in favor of a biofilm lifestyle over a planktonic lifestyle that fosters bacterial spread, thus reducing the systemic colonization of plants (Wallis et al., 2020).

## 14 The global landscape of *Vitis* germplasm response to *Xylella fastidiosa*

In **Tables 2**, **3**, a summary of the grape species and varieties screened for resistance to *X. fastidiosa* has been compiled, respectively, for the subgenera *Euvitis* and *Muscadinia*. A more extensive catalog of the screening efforts, detailing the type of study, the mode of inoculation and infection assessment, the plant response to the infection, as well as the corresponding literature, is reported in **Supplementary Table S1**.

**Table 2:** Summary of the response to *Xylella fastidiosa* infections in genotypes belonging to the subgenus *Euvitis*.

Subgenus *Euvitis*	Species/varieties/selection/genotype	Susceptible	Tolerant/resistant
European species and crossings	*Vitis vinifera* subsp*. vinifera*	79	24
	*Vitis* interspecific crossing	8	51
	Rootstocks	19	1
American species and crossings of Vitis arizonica	*Vitis × doaniana*	1	3
	*Vitis acerifolia*	2	1
	*Vitis aestivalis*	7	7
	*Vitis aestivalis × Vitis vinifera*	0	1
	*Vitis aestivalis* subsp*. smalliana*	1	11
	*Vitis aestivalis* var. *smalliana x Vitis simpsonii*	0	3
	*Vitis shuttleworthii*	0	7
	*Vitis rupestris*	10	3
	*Vitis simpsonii*	0	3
	*Vitis tiliaefolia*	1	3
	*Vitis treleasei*	2	15
	*Vitis berlandieri*	30	11
	*Vitis californica*	2	0
	*Vitis candicans*	0	35
	*Vitis caribaea*	0	1
	*Vitis champinii*	1	4
	*Vitis cinerea*	5	23
	*Vitis cinerea × Vitis berlandieri*	0	1
	*Vitis girdiana*	10	25
	*Vitis labrusca*	8	0
	*Vitis labrusca × Vitis vinifera*	0	1
	*Vitis lincecumii*	0	1
	*Vitis monticola*	3	10
	*Vitis nesbittiana*	0	3
	*Vitis riparia*	19	4
	*Vitis rufotomentosa*	0	2
	*Vitis vulpina*	0	2
	*Vitis arizonica* × *Vitis rupestris* A. de Serres hybrids	0	2
	9622 progeny of D8909-15 × F8909-17 (*Vitis rupestris* A de Serres × *Vitis arizonica/candicans* b43-17) PdR1 locus	4	137
	*Vitis arizonica/candicans* b43-17 × *Vitis. rupestris* A. de Serres	1	8
	*Vitis.s arizonica* × *V. vinifera*	1	1
	*Vitis. arizonica/girdiana*	0	1
	*Vitis. arizonica* (resistance not associated with a PdR1 locus or with unknown parentage)	0	2

Genotypes are distinguished as European and American species and their crosses. The susceptible classification groups the categories of susceptible and highly/moderately susceptible, while the tolerant/resistant classification groups the categories of tolerant, highly/moderately tolerant, and highly/moderately resistant, as well as their combinations, as reported in **Supplementary Table S1**.

**Table 3:** Summary of the response to *X. fastidiosa* infection of genotypes belonging to the subgenus *Muscadinia*, distinguished among its three subspecies.

Subgenus *Muscadinia*	Subspecies	Susceptible	Tolerant/resistant
*Vitis rotundifolia*	Subspecies *rotundifolia*	2	110
	Subspecies *munsoniana*	–	3
	Subspecies *popenoei*	–	1

The susceptible classification groups the categories of susceptible and highly/moderately susceptible, while the tolerant/resistant classification groups the categories of tolerant, highly/moderately tolerant, and highly/moderately resistant, as well as their combinations, as reported in **Supplementary Table S1**.

Genotypes are classified into the two main subgenera *Euvitis* and *Muscadinia* and further categorized based on species and genotypes (**Supplementary Table S1**). The majority (514 out of 627 records) of trials involving *Euvitis* genotypes have been carried out by artificial inoculation, with the host response (resistance/susceptibility) measured based on symptom scoring (554 out of 627 records) and/or bacterial population (503 out of 627 records). For a limited number of genotypes, host response to *X. fastidiosa* infection was assessed based on physiological (xylem hydraulic), biochemical (phenolics, polysaccharides, and xylem sap composition), or microscopic (vessel occlusions, tyloses) observations. Bacterial populations in the tested plants were generally estimated by ELISA (213 out of 627 records), while only 38 out of 627 genotypes were subjected to different PCR techniques and 58 were assessed using chemical (HPLC), immunochemical (FITC), anatomical, and microscopic observations. In some records, combinations of techniques were used. Based on the response to infections, grape genotypes were generally categorized as susceptible, tolerant to resistant, and, in some cases, as moderately/highly susceptible, moderately/highly resistant, or intermediate. Several genotypes were categorized using combinations of different, although similar, classes, while one record had contrasting phenotypes (i.e., Flame Seedless in **Supplementary Table S1** reported as susceptible and resistant in diverse records).

Data analysis confirms the susceptibility of *V. vinifera*; 79 out of 104 (75%) genotypes were classified as susceptible, moderately susceptible, and highly susceptible, while only 24 out of 104 (23%) were reported as tolerant, resistant, highly/moderately resistant, or intermediate. One genotype had contrasting (i.e., susceptible or resistant) responses to the infection. Among the American group of *Euvitis*, 75% (331 out of 439) of genotypes were tolerant, resistant, highly/moderately resistant, or intermediate. These belonged mainly to *V. arizonica* and its 9,622 progeny, which were used to identify the resistance loci or for crosses with *V. vinifera* and *V. rupestris*. All other American species belonging to *Euvitis* were minimally represented, with different phenotypic responses to the infections. Notably, 70% of varieties used as rootstocks were found to be susceptible.

Data analysis strongly points to the known resistance of southeastern US grapes, represented by varieties of *Muscadinia rotundifolia*. More than 98% of records resulted in these genotypes being categorized as tolerant/resistant, with different intermediate levels. Although some varieties were surveyed in multiple trials, their categorizations were consistently confirmed. The majority of records derived from experimental trials (86 out of 111 records) were exposed to natural or artificial inoculation. A smaller number of categorizations (five) were based on direct field observations or bioassays (20). Artificial (58) and natural (53) inoculations were almost equally distributed. The “Categorization of the host response” based on bacterial population level estimated by ELISA or symptom expression was reported in 33 and 65 out of 111 records, respectively, while 35 records were based on chemical (xylem sap composition), anatomical (vessel occlusions, xylem morphology), and molecular (RNA-seq) features. A single study used bacterial culture filtrate infiltration to evaluate the tissue response of 20 muscadine varieties. The other techniques used were chromatography and spectrometry, mainly to investigate sap composition and, in very few studies, electron microscopy.

## 15 Concluding remarks

The analysis of the extensive literature available to date on the grapevine–*X. fastidiosa* interaction indicates that research into grapevine resistance to PD has remarkably increased over the last century, moving from early empirical field and greenhouse observations to recent advanced molecular and genomic investigations. In this review, the multifactorial nature of grapevine responses to the bacterium has been emphasized, demonstrating that PD resistance is a quantitative trait resulting from the integration of multiple defense strategies operating at different biological levels.

The different mechanisms proposed to underlie grapevine resistance to PD are schematically summarized in **Figure 1**.

**Figure 1:**
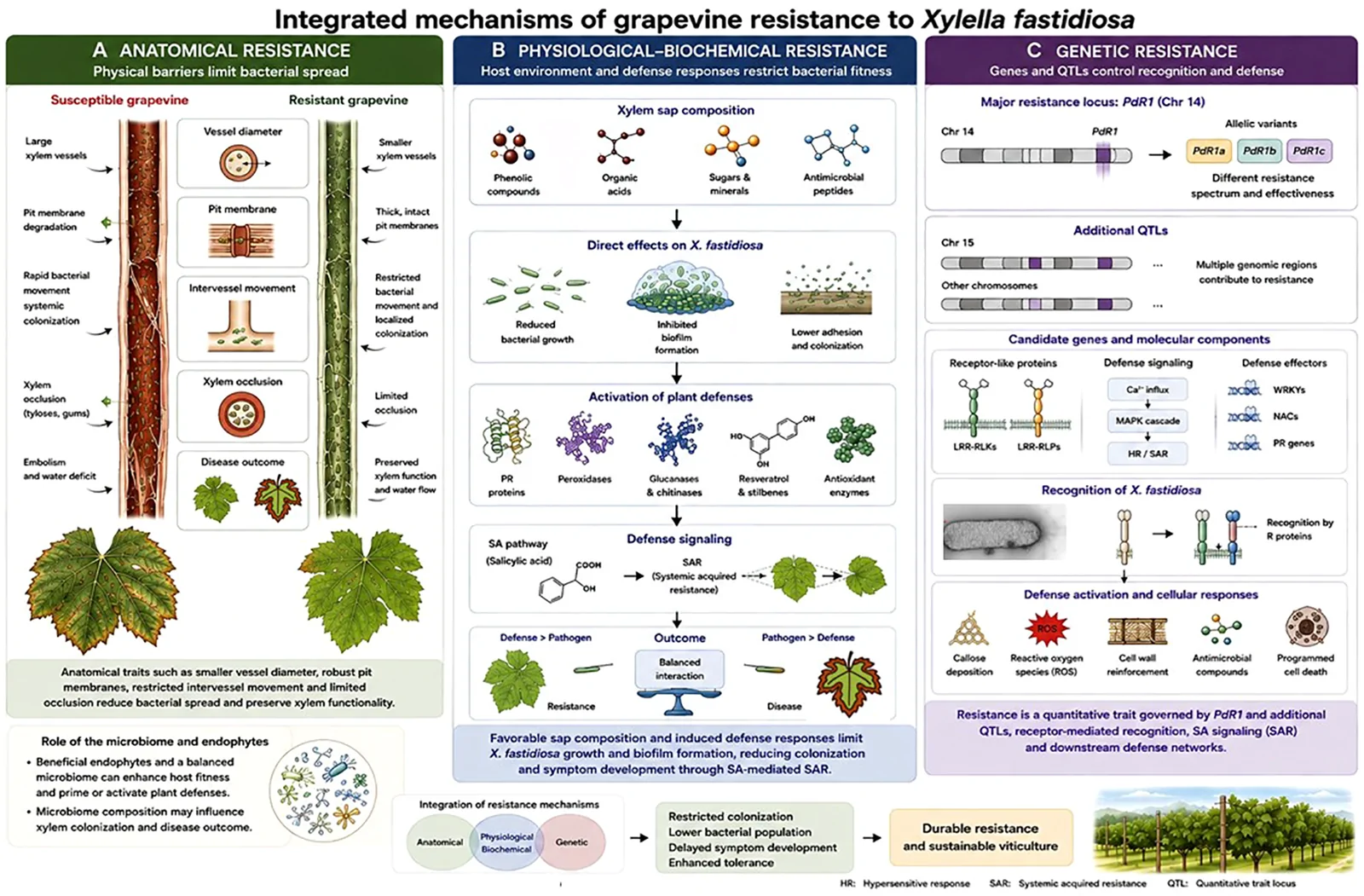
Cartoon representation of the integrated mechanisms underlying grapevine resistance to *Xylella fastidiosa*. The multifaceted mechanisms of resistance are depicted, relying on: anatomical restrictions of the bacterial spread **(A)**, physiobiochemical reactions to bacterial infections **(B)**, and genetic recognition of the bacterium or plant constituents to mount a defense response **(C)**. Resistance is based on the integration of these three mechanisms. Components of each mechanism and their role are detailed in the modules.

From the earliest reports of “Anaheim disease” in California and the parallel observations in the southeastern USA, it became increasingly evident that susceptibility and resistance to PD were not randomly distributed among the genus *Vitis*. Indeed, native American grape species, particularly those originating from regions historically exposed to high natural PD pressure, consistently exhibited reduced symptom severity and enhanced survival compared to *V. vinifera* varieties, which in contrast collapsed within a few years after planting. This geographical pattern strongly suggests that resistance traits emerged through a long evolutionary coexistence between grape hosts, insect vectors, and the pathogen. These pioneering observations laid the foundation for decades of breeding and physiological research aimed at understanding the host determinants of resistance and incorporating these into *V. vinifera* germplasm.

The identification of *X. fastidiosa* as the etiological agent of PD represented a major turning point, accelerating large-scale screening activities by standardizing inoculation and phenotyping protocols and enabling the development of reliable diagnostic tools. The evaluation of hundreds of grape genotypes under both greenhouse and field conditions has highlighted the strong relationship between bacterial population dynamics, symptom progression, and host response.

Electron microscope observations revealed ultrastructural alterations occurring in the xylem vessels, demonstrating that susceptible grapevines generally exhibit a greater extent of vessel occlusion and tylose formation than resistant genotypes, which generally display lower vessel colonization, reduced hydraulic impairment caused by the formation of emboli, and more localized infections. Physiological studies have shown that anatomical and biochemical features, including vessel diameters, pit membrane composition, and vessel connectivity, strongly influence resistance or susceptibility to PD. Moreover, the interaction between bacterial CWDEs and intervessel PMs appears to play a central role in disease progression. PMs of susceptible varieties appeared to be more vulnerable to enzymatic degradation by *X. fastidiosa*, facilitating bacterial movement between vessels and resulting in severe hydraulic conductivity losses, extensive vessel occlusion, and enhanced symptom severity. Conversely, resistant genotypes can better preserve xylem integrity against CWDEs, preventing systemic bacterial spread.

Over decades, extensive surveys, field trials, and controlled inoculation experiments have identified muscadines and several southeastern US wild grape species as major reservoirs of resistance-associated traits.

The discovery and mapping of the *PdR1* locus in one of these native species, *V. arizonica*, represented the most important milestone in PD grapevine resistance research. The characterization of this locus, together with the development of linked molecular markers, greatly improved the efficiency of breeding programs by enabling MAS for the early selection of resistant seedlings, thereby accelerating the incorporation of resistance traits into elite *V. vinifera* backgrounds.

The grape breeding program at UC Davis has recently released PD-resistant varieties, including “Camminare”, “Passeante Noir”, “Errante Noir”, “Ambulo Blanc”, and “Caminante Blanc”. However, in addition to taking a long time and incurring costs, classical plant breeding, based on crossing with wild parents, suffers from the drag of undesired traits transferred along with the target gene of resistance, which could affect the quality of the grape. Therefore, targeted biotechnological and transgenic approaches acting at different stages of the infection process have proved effective in reducing susceptibility to PD. Strategies based on the inhibition of bacterial CWDEs, delivery of antimicrobial peptides, manipulation of quorum-sensing signaling, and the transfer of resistance-related genes from wild grape species consistently resulted in reduced bacterial populations and symptom severity under controlled conditions. Although further validation under long-term field conditions is still required, these studies demonstrate the potential of biotechnology as a complementary tool to conventional breeding for sustainable PD management.

More recently, the integration of high-resolution genetic mapping, the availability of phased genome assemblies, and the application of omics technologies (proteomics, metabolomics, transcriptomics, and functional genomics) have considerably advanced the characterization of grapevine–*Xylella* interactions and the molecular basis of resistance. These tools have demonstrated that PD resistance is not exclusively controlled by the *PdR1* locus, as additional QTLs and candidate resistance genes have been identified in several wild *Vitis* species, providing new opportunities for gene pyramiding strategies and the development of more durable resistance in grape breeding programs. In particular, the application of transcriptomic and functional genomics approaches highlighted that resistant and susceptible genotypes differ not only in the activation of defense-related genes but also in the regulation of metabolic, respiratory, and signaling pathways. Recent research in functional genomics has uncovered novel components involved in grapevine resistance, including respiratory homeostasis, host–microbiome interactions, and long noncoding RNA-mediated regulation. Taken together, these studies have expanded the traditional view of resistance beyond pathogen recognition and defense activation, confirming that resistance involves extensive transcriptional reprogramming and metabolic adaptation. The composition of grapevine xylem sap strongly influences bacterial growth, biofilm formation, and aggressiveness of the strains, with saps from susceptible varieties generally promoting greater bacterial multiplication, whereas those from resistant genotypes tend to suppress these processes. Moreover, lower levels of phenolic compounds and other secondary metabolites were frequently observed in resistant grapevines. Therefore, the biochemical profile of the sap could modulate bacterial lifestyle by favoring biofilm formation over systemic spread and consequently reducing plant colonization. Proteomic investigations identified the recurrent accumulation of numerous defense-related proteins, including pathogenesis-related proteins, glucanases, chitinases, and peroxidases, as well as proteins involved in cell-wall remodeling and stress responses, thereby facilitating the identification of candidate proteins potentially associated with resistance and involved in the infection process.

An overview of the major milestones that have shaped our current understanding of PD resistance over the last century is depicted in **Figure 2**.

**Figure 2:**
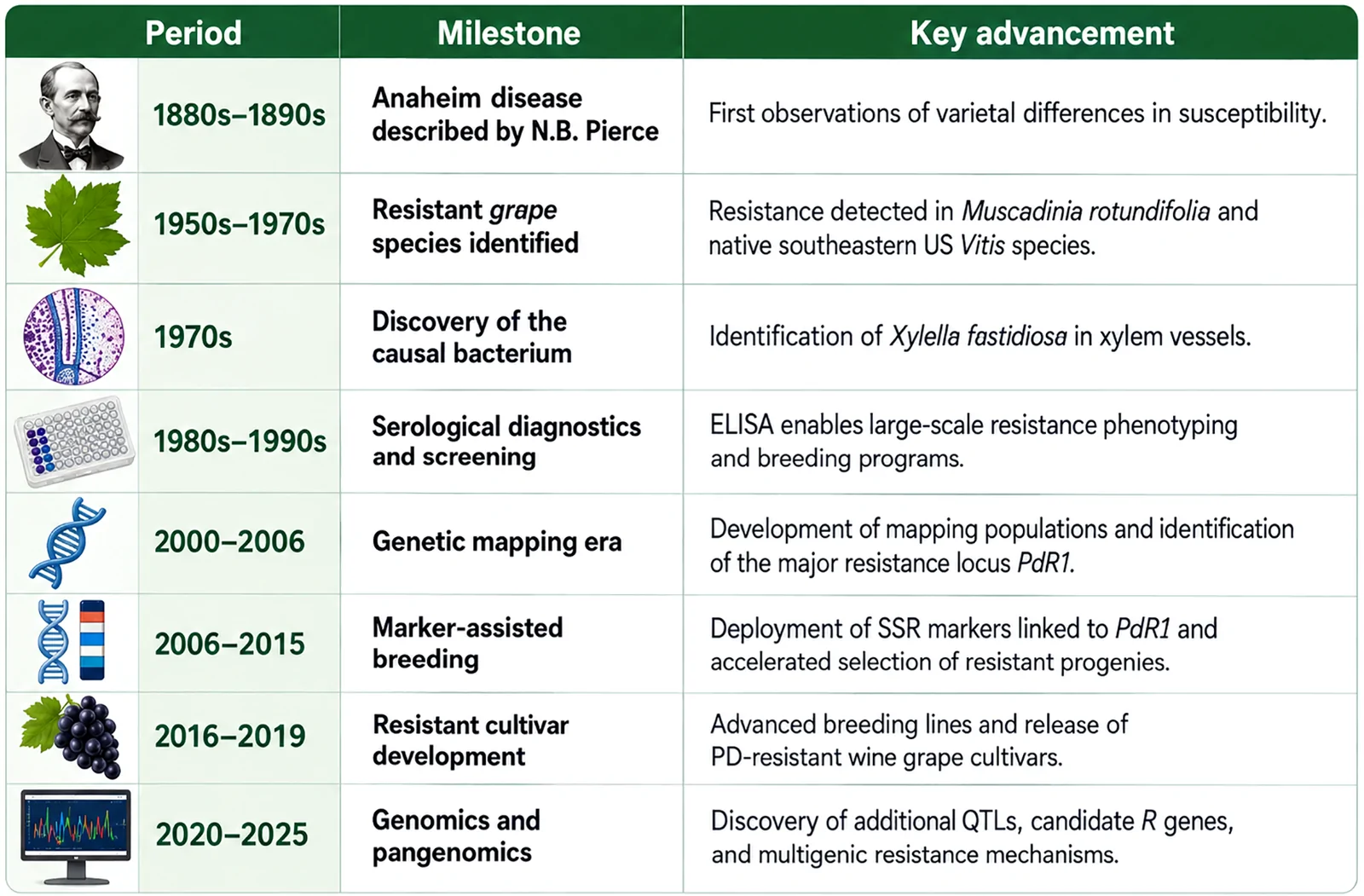
Historical timeline of the major milestones in PD resistance research in grapevine over the last century. The cartoon illustrates the major milestones achieved in the study of PD resistance and their contributions to the advancement of resistance mechanisms and breeding.

Despite the remarkable progress achieved so far, several aspects of PD resistance remain only partially understood. Important questions still concern the durability of resistance loci under different environmental conditions, the contribution of minor-effect genes, the interaction with the host genotype genetic background, the role of endophytic microbial communities, and the impact of climatic variables on disease severity and vector ecology.
