# Correction: Genetic and physiological mechanisms of grapevine resistance to *Xylella fastidiosa* infection: a progressive acquisition of knowledge

**DOI:** 10.3389/fpls.2026.2008052

**Published:** 2026-10-19

**Authors:** Serafina Serena Amoia, Pasquale Saldarelli

**Affiliations:** Consiglio Nazionale delle Ricerche, Istituto per la Protezione Sostenibile delle Piante (CNR-IPSP), Bari, Italy

**Keywords:** biotechnology, breeding, grapevine, MAS, Pierce’s disease, resistance, *Xylella fastidiosa* subsp. *fastidiosa*

The **Generative AI statement** was erroneously given as “The author(s) declared that generative AI was used in the creation of this manuscript. **Figure 1** and **2** were generated by AI. Any alternative text (alt text) provided alongside figures in this article has been generated by Frontiers with the support of artificial intelligence and reasonable efforts have been made to ensure accuracy, including review by the authors wherever possible. If you identify any issues, please contact us.”

The correct **Generative AI statement** is “The author(s) declared that **Figure 1** and **2** were generated by AI.

Any alternative text (alt text) provided alongside figures in this article has been generated by Frontiers with the support of artificial intelligence and reasonable efforts have been made to ensure accuracy, including review by the authors wherever possible. If you identify any issues, please contact us.”

Several corrections have also been made to the body of the article to correct minor grammatical and typographical errors.

A correction has been made to section *6.1*, paragraph 2

“We need to look back to 1961, when Harold P. Olmo, a grape breeder and professor at the University of California, Davis, collected 85 genotypes during a trip to Mexico, where PD is also endemic, including a few clones of V. arizonica (Vasquez, 2006). Following the experiences of colleagues in the southeastern USA, Olmo crossed female *V. rupestris* with male *V. rotundifolia* individuals, obtaining an F1 population, named “89”, that showed strong resistance to both the dagger nematode *Xiphinema index* (the vector of Grapevine fanleaf virus) and PD, with both traits segregating as single loci. To genetically characterize the locus carrying the resistance trait, Walker, who inherited the “89” group, crossed two siblings, D8909–15 and F8909-17, to generate an F2 population later named “9621” (Doucleff et al., 2004). Successive studies using single sequence repeats (SSRs) markers and morphological features (i.e., neither *V. rupestris* nor *V. rotundifolia* has hairs on their stems or leaves, and their progeny should be sterile, which was not the case for the 89 and 9621 populations) revealed that: (1) D8909–15 and F8909–17 were not siblings; (2) most of the resistant “89” seedlings originated from *V. rupestris* females unintentionally pollinated by *V. arizonica* and its hybrids with *V. candicans*; and (3) D8909–15 and F8909–17 were, respectively, interspecific hybrids of *V. rupestris* “A. de Serres” × b42-26 (a *V. arizonica* selection from Baja California, Mexico) and *V. rupestris* “A. de Serres” × b43-17 (a *V. arizonica* from Monterrey, Mexico) (Riaz et al., 2007). Therefore, only very few seedlings had the *V. rupestris* or *V. rotundifolia* parentage that Olmo intended to obtain. Nevertheless, the unexpected discovery of the PD resistance in *V. arizonica* prompted Walker and collaborators to direct future investigations toward this grape species.”

A correction has been made to Section *6.3*, paragraph 2:

“The genotype IAC-572, a *V. caribaea* hybrid rootstock, manifested very few PD symptoms with both strains, albeit harboring high bacterial populations when inoculated with isolate N-001, thus representing a promising source of resistance.”

A correction has been made to section *7.3*, paragraph 5:

“The most distinct signals were detected on chromosomes 14 and 15.”

A correction has been made to the section **11**:

“Using this combined strategy, they detected 91 proteins in the xylem sap from infected and noninfected plants; in particular, this approach allowed them to recognize increased levels of proteins linked to the defense response in infected plants, such as PR proteins (PR-1 and β-1,3-glucanases) alongside proteins involved in the cell-wall/vascular remodeling (chitinases, expansin, lipid-transfer protein), including a blue copper protein-like protein involved in lignin deposition.”

A correction has been made to the section **14**, paragraph 2:

“Data analysis confirms the susceptibility of V. vinifera; 79 out of 104 (75%) genotypes were classified as susceptible, moderately susceptible, and highly susceptible, while only 24 out of 104 (23%) were reported as tolerant, resistant, highly/moderately resistant, or intermediate. One genotype had contrasting (i.e., susceptible or resistant) responses to the infection. Among the American group of *Euvitis*, 75% (331 out of 439) of genotypes were tolerant, resistant, highly/moderately resistant, or intermediate.”

A correction has been made to section **15**; paragraph 9:

“More recently, the integration of high-resolution genetic mapping, the availability of phased genome assemblies, and the application of omics technologies (proteomics, metabolomics, transcriptomics, and functional genomics) have considerably advanced the characterization of grapevine–*Xylella* interactions and the molecular basis of resistance.”

**Supplementary Table 2** was also mistakenly included in the original publication and has now been removed.

The original version of this article has been updated.

